# Helicusin E, Isochromophilone X and Isochromophilone XI: New Chloroazaphilones Produced by the Fungus *Bartalinia robillardoides* Strain LF550

**DOI:** 10.3390/md11030800

**Published:** 2013-03-12

**Authors:** Nils Jansen, Birgit Ohlendorf, Arlette Erhard, Torsten Bruhn, Gerhard Bringmann, Johannes F. Imhoff

**Affiliations:** 1 Kiel Centre for Marine Natural Products at the Helmholtz Centre for Ocean Research (GEOMAR), Kiel 24106, Germany; E-Mails: njansen@geomar.de (N.J.); bohlendorf@geomar.de (B.O.); aerhard@geomar.de (A.E.); 2 Institute of Organic Chemistry, University of Würzburg, Am Hubland, Würzburg 97074, Germany; E-Mails: bruhn@chemie.uni-wuerzburg.de (T.B.); bringman@chemie.uni-wuerzburg.de (G.B.)

**Keywords:** marine natural product, azaphilone, pentaketide, *Bartalinia robillardoides*, *Tethya aurantium*

## Abstract

Microbial studies of the Mediterranean sponge *Tethya aurantium* led to the isolation of the fungus *Bartalinia robillardoides* strain LF550. The strain produced a number of secondary metabolites belonging to the chloroazaphilones. This is the first report on the isolation of chloroazaphilones of a fungal strain belonging to the genus *Bartalinia*. Besides some known compounds (helicusin A (**1**) and deacetylsclerotiorin (**2**)), three new chloroazaphilones (helicusin E (**3**); isochromophilone X (**4**) and isochromophilone XI (**5**)) and one new pentaketide (bartanolide (**6**)) were isolated. The structure elucidations were based on spectroscopic analyses. All isolated compounds revealed different biological activity spectra against a test panel of four bacteria: three fungi; two tumor cell lines and two enzymes.

## 1. Introduction

Filamentous fungi represent an important group of microorganisms known for effective production of secondary metabolites. In nature these compounds play a role e.g., in communication pathways or as a defense against other microorganisms. On top of the advantageous effects for their producers, fungal secondary metabolites often show considerable affinity to mammalian targets resulting in pharmaceutically relevant bioactivities. Hence, these metabolites can be used for human benefit as they have a potential to be used as drugs [1]. Strains of the genus *Bartalinia robillardoides* have been shown to produce paclitaxel, an anticancer drug already clinically applied [2]. Our reason for selecting a *Bartalinia robillardoides* strain (LF550) for a detailed study of its secondary metabolite profile was the wide range of produced secondary metabolites detected in the HPLC chromatogram of the fungal crude extract. In preliminary experiments strain LF550 was isolated from the marine sponge *Tethya aurantium* in contrast to other known *Bartalinia robillardoides* strains, including the paclitaxel producer, which are reported to be endophytic [2]. Besides known compounds, which are uncommon for this genus, we identified four new metabolites in the metabolite spectrum of this strain. Most of the isolated compounds could be classified as members of the azaphilone family. Azaphilones are typified by an oxygenated 7-hydroxy-7-methyl-isochromene-6,8-dione skeleton [3] and by a 4*H* pyran ring with a high affinity to primary amines [4]. Azaphilones comprise pigments and a variety of molecules with different bioactivities [5].

## 2. Results and Discussion

*Bartalinia robillardoides* strain LF550 was isolated from the marine sponge *Tethya aurantium*, originated from the Limsky kanal (Canal di Lemme or Limsky channel, Croatia), a small fjord in the Mediterranean. The strain was taxonomically characterized by sequence analysis of the internal spacer region (ITS), which revealed 99% similarity to *Bartalinia robillardoides* CBS:122686 (NCBI™ Accession No. EU552102). Morphological studies supported this classification and revealed conidia with characteristic structures of the genus *Bartalinia* [2]. It was the first time that a fungus of the genus *Bartalinia* was isolated from a marine sponge [6].

For production of secondary metabolites the strain was grown in 10 L of a malt-extract medium at 22 °C. After 21 days the culture broth was extracted. After purification and column chromatography, spectral analysis of the compounds enabled the identification of the known compounds helicusin A [7] (**1**) and deacetylsclerotiorin [8] (**2**) by comparison with literature data.

Azaphilones represent a widespread family of fungal pigments. A quite recent comprehensive review by Osmanova *et al*. (2010) includes more than 170 azaphilones that are produced by 23 different genera from 13 fungal families [5]. Therefore, the detection of azaphilones in a fungal extract is not surprising. However, production of azaphilones has not yet been described for the genus *Bartalinia*. In particular, the chlorinated congeners are less common. Their production has so far been described only for five other fungal genera, *Penicillium*, *Chaetomium*, *Emericella*, *Talaromyces* and *Fusarium* [5].

**Figure 1:**
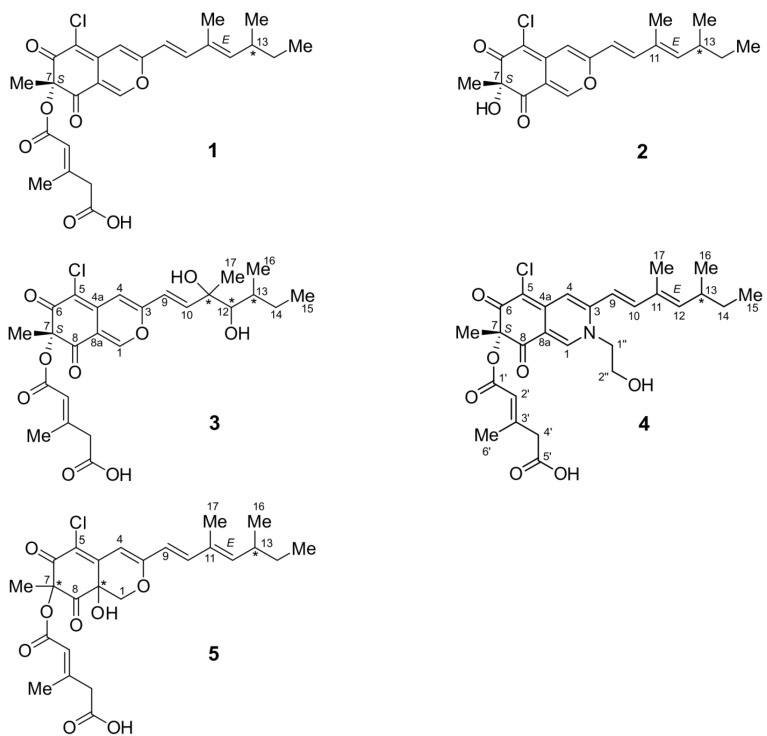
Structures of the compounds **1**–**5**.

In addition to these known compounds, four new compounds were identified (Figure 1): the chloroazaphilone derivatives helicusin E (**3**), isochromophilone X (**4**) and isochromophilone XI (**5**) as well as bartanolide (**6**), a ten-membered lactone with a crotonic acid side chain. 

### 2.1. Structure Elucidation

#### 2.1.1. Isochromophilone X (**4**)

The molecular formula C_27_H_33_N^35^ClO_7_ of **4** was determined by high-resolution ESI-MS ([M + H^+^] *m/z* 518.1940, calcd. for C_27_H_33_N^35^ClO_7_: *m/z* 518.1940). The isotope pattern observed in the mass spectrum confirmed that the molecule contained one chlorine atom. The number of carbons was in accordance with 27 individual signals detected in the ^13^C-NMR spectrum. The ^1^H–^13^C HSQC experiment enabled us to assign all of the hydrogen signals to the signals of their directly bonded carbons (Table 1). We identified five methyl groups, four methylene groups, six olefinic methine groups and one alkyl methine group, so that eleven quaternary carbons (Table 1) remained. Four of them were carbonyl quaternary carbons, six were olefinic carbons and one was a quaternary carbon connected to an oxygen atom. It became evident that **4**, just like **1**–**3**, was a chloroazaphilone, its planar structure was determined mainly based on the ^1^H–^13^C HMBC data. In comparison to NMR data of some related structures [7,9,10], our data indicated a similar isoquinoline-6,8-dione skeletal structure substituted with a 3,5-dimethyl-1,3-heptadienyl side chain. Another side chain, a (2*E*)-3-methylpent-2-enedioic acid, was linked by an ester bond as found in the helicusins and a hydroxyethyl moiety as described for isochromophilone VI [9].

**Table 1:** NMR-spectroscopic data (500 MHz, methanol-*d*_4_) for isochromophilone X (**4**).

Position	δ_C_, type	δ_H_ (*J* in Hz)	COSY	HMBC	NOESY
1	144.5, CH	8.17, s		3, 5, 4a, 8, 8a, 1″	1″
3	151.9, C				
4	112.6, CH	7.20, s		3, 5, 8, 8a, (9)	
4a	148.3, C				
5	116.4, C				
6	185.7, C				
7	85.7, C				
8	195.2, C				
8a	101.4, C				
9	117.3, CH	6.58, d (15.5)	10	4, 10, 11, 12	17, 1″
10	146.4, CH	7.11, d (15.5)	9	3, 9, 11, 12, 17	12
11	133.9, C				
12	148.7, CH	5.78, d (9.6)	13, (17)	10, 13, 14, 17, 16	10, 14b, 16
13	36.2, CH	2.54, m	12, 14, 16	11, 12, 14, 16, 15	14a, 16, 17
14a	31.2, CH_2_	1.47, m	13, 15	12, 13, 15, 16	13
14b		1.36, m	13, 15	12, 13, 15, 16	12
15	12.4, CH_3_	0.90, t (7.4)	14	13, 14	
16	20.6, CH_3_	1.04, d (6.7)	13	12, 13, 14	12, 13
17	12.7, CH_3_	1.92, d (1.0)	(12)	10, 11, 12, (14), (16)	9, 13
18	23.7, CH_3_	1.52, s		6, 7, 8	
1′	166.3, C				
2′	118.7, CH	5.97, d (1.1)	(6′)	1′, 3′, 4′, 6′	4′
3′	156.1, C				
4′	46.6, CH_2_	3.21, s		2′, 5′, 6′	2′, 6′
5′	173.4, C				
6′	19.3, CH_3_	2.15, d (1.2)	(2′)	1′, 2′, 3′, 4′, 5′	4′
1″	57.4, CH_2_	4.24, d (4.4)	2″	1, 3, 2″	1, 9
2″	61.1, CH_2_	3.85, t (5.2)	1″	1″	

The 3,5-dimethyl-1,3-heptadienyl side chain of **4** included two separated spin systems. The first one consisted of two methyl groups [CH_3_-15 (δ_H_ 0.90, δ_C_ 12.4) and CH_3_-16 (δ_H_ 1.04, δ_C_ 20.6)], one methylene group [CH_a_-14 (δ_H_ 1.47, δ_C_ 31.2)/CH_b_-14 (δ_H_ 1.36, δ_C_ 31.2)] and two methine groups [CH-13 (δ_H_ 2.54, δ_C_ 36.2) and CH-12 (δ_H_ 5.78, δ_C_ 148.7)]. The COSY experiment revealed two important results: (1) CH_3_-15 was adjacent to CH_2_-14 and (2) CH-13 coupled to CH_2_-14, to the methyl carbon CH_3_-16, and to the olefinic methine carbon CH-12. This first spin system was connected via the quaternary carbon C-11 (δ_C_ 133.9) to the second spin system of the partial structure consisting of the two olefinic carbons CH-10 (δ_H_ 7.11, δ_C_ 146.4) and CH-9 (δ_H_ 6.58, δ_C_ 117.3). CH-9 joined the side chain to the isoquinolin-6,8-dione skeletal structure via the quaternary carbon C-3 (δ_C_ 151.9). A third methyl group CH_3_-17 (δ_H_ 1.92, δ_C_ 12.7) was bonded to C-11 as evidenced by the ^1^H–^13^C HMBC correlation from C-17 to C-10 and to C-12. These data evidenced the structure of the 3,5-dimethyl-1,3-heptadienyl side chain.

The shifts of the olefinic carbons CH-1 (δ_H_ 8.17, δ_C_ 144.5) and C-3 (δ_C_ 151.9) indicated the presence of a nitrogen atom adjacent to them. CH-1 was correlated to C-3 via a long-range ^1^H–^13^C HMBC coupling which was transmitted via the nitrogen atom. Moreover H-1 showed long-range couplings to C-8a (δ_C_ 101.4), C-4a (δ_C_ 148.3) and C-8 (δ_C_ 195.2), which were valuable data for the identification of the quaternary carbons that interconnect the two rings of the isoquinoline-6,8-dione substructure. The ^1^H–^13^C HMBC correlations of the olefinic proton signal CH-4 supported the delineated structure and the long-range coupling to CCl-5 made this the most probable site for chlorination, which was in perfect agreement with known chloroazaphilones. The following data completed the isoquinoline-6,8-dione skeleton [7,8]. The low-field shift of C-8 and C-6 (δ_C_ 185.7) proved them both to be ketone carbonyl carbons. The ^1^H–^13^C HMBC couplings from CH_3_-18 (δ_H_ 1.52, δ_C_ 23.7) gave evidence of its proximity to both carbonyl carbons C-6 and C-8 as well as an oxygenated carbon C-7 (δ_C_ 85.7).

The ^1^H–^13^C HMBC coupling of CH_2_-1″ (δ_H_ 4.24, δ_C_ 57.4) to CH-1 and to C-3 proved CH_2_-1″ to be linked to the isoquinoline-6,8-dione core structure. The chemical shift of C-1″ confirmed the bondage to a nitrogen atom. The COSY experiment proved the two methylene groups CH_2_-1″ and -CH_2_-2″ (δ_H_ 3.85, δ_C_ 61.1) to form a small spin system. As evidenced by its chemical shift CH_2_-2″ was substituted with a hydroxy group. These data unambiguously determined the presence of a 2-hydroxyethyl side chain.

The branched ester moiety consisted of one methyl group CH_3_-6′ (δ_H_ 2.15, δ_C_ 19.3), a carboxylic carbon C-5′ (δ_C_ 173.4), a methylene group CH_2_-4′ (δ_H_ 3.21, δ_C_ 46.6), a quaternary carbon C-3′ (δ_C_ 156.1), an olefinic methine group CH-2′ (δ_H_ 5.97, δ_C_ 118.7) and an ester carbonyl carbon C-1′ (δ_C_ 166.3). None of the protons of this partial structure coupled with each other so the delineation of the structure mainly relied on the ^1^H–^13^C HMBC data. The correlation of the methylene group CH_2_-4′ to the carboxylic carbon C-5′ on the one hand and to the quaternary carbon C-3′ and the olefinic methine carbon CH-2′ on the other hand, established the sequence from C-5′ to CH-2′. CH-2′ was linked to the carboxylic carbons C-1′ and C-3′ as shown by its long range couplings. Finally, the olefinic methyl group CH_3_-6′ was bonded to C-3′. Due to its central position in this side chain, ^1^H–^13^C HMBC couplings to all other carbons of the side chain (C-1′, C-2′, C-3′, C-4′ and C-5′) could be observed. Thus, the ester moiety was proven to be a (2*E*)-3-methyl-2-pentenedioyl (*trans*-3-methylglutaconyl) residue. The most plausible position of this side chain was C-7. This assumption was confirmed by the chemical shift of C-7 (δ_C_ 85.7).

To sum up the aforementioned data, a 2*H*-isoquinoline-6,8-dione skeleton was evident, possessing the constitution of **4** with all side chains as shown in Figure 1.

Wei and Yao [4] reported that highly oxygenated chloroazaphilones, such as helicusin A, react with small primary amines within a few minutes. Therefore, isochromophilone X might be a product of a reaction between helicusin A and the primary amine 2-aminoethanol. This was shown in an *in vitro* assay by Wei and Yao [4]: Accordingly, **1** was dissolved in dichloromethane (DCM) and 2-aminoethanol was added. The color change from yellow to red was observed within a minute. After 5 min, the yield was approximately 98%. To check whether the medium contained 2-aminoethanol, **1** was solved in freshly prepared malt extract medium. After 24 h, the mixture was extracted and analyzed. In the HPLC-DAD/MS spectra, neither the characteristic UV/VIS absorptions nor the signal in the MS spectrometer could be seen for the conversion to **4**.

#### 2.1.2. Isochromophilone XI (**5**)

The molecular formula C_25_H_30_^35^ClO_8_ of **5** was determined by high-resolution ESI-MS ([M + H^+^] *m/z* 493.1625, calcd. for C_25_H_30_^35^ClO_8_: *m/z* 493.1624). The isotope pattern observed in the mass spectrum confirmed that the molecule contained one chlorine atom. The compound was found to contain three known structural units but proved to be a new derivative of isochromophilone VII. The NMR data of the skeleton and the 3,5-dimethyl-1,3-heptadienyl side chain were in agreement with those described in literature [11]. The additional signals of the (2*E*)-3-methyl-2-pentenedioyl (*trans*-3-methylglutaconyl) residue matched with those reported earlier [7].

#### 2.1.3. Helicusin E (**3**)

The molecular formula C_25_H_30_N^35^ClO_9_ of **3** was determined by high-resolution ESI-MS ([M+H^+^] *m/z* 509.1585, calcd. for C_25_H_30_N^35^ClO_9_: *m/z* 509.1573). The isotope pattern observed in the mass spectrum confirmed that the molecule contained one chlorine atom. The mass difference between helicusin A and helicusin E was 34, which indicated the possible presence of two hydroxy groups. The signals of the (2*E*)-3-methyl-2-pentenedioyl (*trans*-3-methylglutaconyl) residue and most of the skeleton matched with those of helicusin A. The high-field shifts of CH-12 δ_H_ 3.49) and CH_3_-17 δ_H_ 0.89) lead to the conclusion that two hydroxy groups as a vicinal diol are attached to C-11 and C-12.

#### 2.1.4. Bartanolide (**6**)

**Figure 2:**
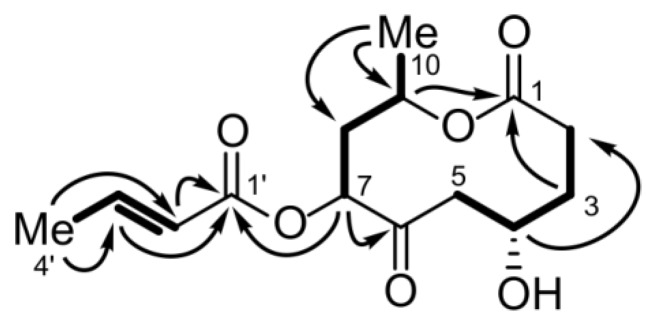
Selected HMBC (arrows) and COSY correlations (bold) for the structure elucidation of **6**.

Bartanolide (**6**) showed structural similarities to other small polyketides like acetoxymultiplolide [12] or achaetolide [13] and hence belonged to a different substance class than the aforementioned azaphilones. The molecular formula C_14_H_20_O_6_ of **6** was confirmed by HRESI/MS. The formula was consistent with the NMR spectral data including the appearance of 14 distinct signals in the ^13^C NMR spectrum (Table 2). Ten of the carbons formed a ten-membered lactone ring with a methyl group next to the ester link. The ^1^H–^1^H COSY spectrum revealed the presence of three separated spin systems, two of which were part of the lactone ring and one belonged to a side chain. The lactone ester carbonyl carbon C-1 (δ_C_ 173.5) was located adjacent to a methylene group CH_2_-2 (δ_C_ 28.5, δ_H_ 2.58 and 2.38), which was part of the spin system reaching from CH_2_-2 to CH_2_-5 (δ_C_ 42.8, δ_H_ 2.82 and 2.28). The spin system consisted of three methylene groups and one oxygen-bearing methine group, CH-4 (δ_C_ 66.2, δ_H_ 4.38). This first spin system was connected to a second one composed of the protons H-7 to H_3_-10 via the carbonyl C-6 (δ_C_ 209.4). The low-field shift of C-6 proved it to be a ketone carbon. Its function as a bridge between the two spin systems was evidenced by ^1^H–^13^C HMBC correlations (Figure 2), *i.e.*, protons from both spin systems, H-5, H-7 and H-8, showed long range couplings to C-6. The second spin system included a methyl group, CH_3_-10 (δ_C_ 20.1, δ_H_ 1.24), one methylene group, CH_2_-8 (δ_C_ 40.5, δ_H_ 2.35 and 2.18) and two oxygen bearing methines, CH-7 (δ_C_ 77.0, δ_H_ 5.02) as well as CH-9 (δ_C_ 67.9, δ_H_ 5.28). The ^1^H–^13^C HMBC correlation of H-9 to C-1 showed that the attached oxygen belonged to the lactone ester group. In analogy to that, H-7 showed a long-range coupling to C-1′ (δ_C_ 167.0), another ester carbonyl carbon. Thus, CH-7 had to be the site where the side chain was linked to the lactone ring. The side chain was proven to be a crotonic acid residue, which consisted of the aforementioned ester group, a double bond, CH-2′ (δ_C_ 122.7, δ_H_ 6.00) and CH-3′ (δ_C_ 148.5, δ_H_ 7.15), and a terminal methyl group CH_3_-4′ (δ_C_ 18.3, δ_H_ 1.96). The high ^3^*J* value between H′-2 and H′-3 of 15.6 Hz indicated the presence of an *E*-configured double bond. Hence, the constitution of the molecule was delineated.

**Table 2:** NMR-spectroscopic data (500 MHz, methanol-*d*_4_) for bartanolide (**6**).

Position	δ_C_, type	δ_H_ (*J* in Hz)	COSY	HMBC	NOESY
1	173.5, C				
2a	28.5, CH_2_	2.58, ddd (18.2, 6.3, 2.3)	3	1, 3, 4	2b, 3b, 3a, 5a
2b		2.38, ddd (18.2, 12.8, 1.8)			2a, 3b, 3a
3a	27.8, CH_2_	2.20, dddd (15.5, 12.8, 2.3, 2.3)	2, 4	1, 2, 5	2a, 2b, 3b, 4
3b		1.68, dddd (15.5, 6.3, 4.5, 1.8)			3a, 4
4	66.2, CH	4.38, dddd (11.5, 4.5, 4.5, 2.3)	3, 5	2, 5	3a, 3b, 5a, 5b
5a	42.8, CH_2_	2.82, dd (18.3, 11.5)	4	3, 4, 6	2a, 4, 5b, 9
5b		2.28, dd (18.3, 4.5)			5a, 4
6	209.4, C				
7	77.0, CH	5.02, dd (6.0, 1.8)	8	6, 8, 9, 1′	8a, 8b
8a	40.5, CH_2_	2.35, ddd (14.6, 12.5, 1.8)	7, 9	6	7, 8b, 9, 10
8b		2.18, ddd (14.6, 6.0, 2.4)			7, 9
9	67.9, CH	5.28, ddq (12.5, 6.5, 2.4)	8, 10	1, 7, 8, 10	3′, 5a, 8a, 8b, 10
10	20.1, CH_3_	1.24, d (6.5)	9	8, 9	8a, 8b, 9
1′	167.0, C				
2′	122.7, CH	6.00, dq (15.6, 1.8)	3′, 4′	1′, 4′	3′, 4′, 5a
3′	148.5, CH	7.15, dq (15.6, 7.0)	2′, 4′	1′, 2′, 4′	5a, 9, 2′, 4′
4′	18.3, CH_3_	1.96, dd (1.8, 7.0)	2′, 3′	2′, 3′	2′, 3′

In summary, the production of bartanolide and a large number of chloroazaphilones provides evidence for the profound biosynthetic potential of *Bartalinia robillardoides*.

#### 2.1.5. Elucidation of the Absolute Configurations of **1–6**

From the literature [7,14,15], it is known that the absolute configuration of the stereocenter at C-7 of compounds **1** and **2** can be deduced from the CD effect at around 390 nm. The stereocenter at C-13, however, has no impact on the CD and is thus out of the scope of such measurements. It is also literature known that the double bond between C-11 and C-12 in the alkyl side chain is configurationally not stable and can undergo an *E*/*Z*-isomerization [16]. The *Z* isomers can be found in traces in the NMR for compounds **1**, **2**, **4**, and **5** already directly after the isolation. Upon standing at room temperature more and more of the *Z* isomers can be observed. Thus, we performed online HPLC-CD investigations to obtain the CD spectra of the pure compounds **1** and **2**. These measurements showed that the *E*- and *Z*-configuration does not significantly influence the CD curve (see SI) and that helicusin A (**1**) and deacetylsclerotiorin (**2**) have a negative Cotton effect at 390 nm, and thus have the *S*-configuration at C-7 as shown in Figure 1.

**Figure 3:**
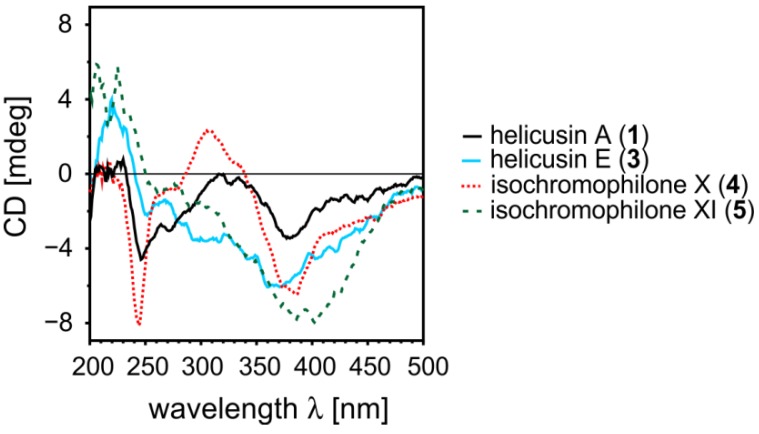
CD spectra of helicusin A (**1**), helicusin E (**3**), and isochromophilone X (**4**) and XI (**5**).

Helicusin E (**3**) has a chromophore that is quite comparable to the ones of **1** and **2** and the negative Cotton effect of this compound at 390 nm (Figure 3) determines the absolute configuration of the stereocenter at C-7 as *S*. In case of the isochromophilone XI (**5**) the chromophore is not comparable to the one of the above mentioned helicusins due to the presence of an additional stereocenter at C-8a. Although the CD spectrum is quite similar to the ones of structures **1**–**3** no reliable conclusion on the absolute configuration at C-7 can be drawn [17]. The high conformational flexibility of **5** (several thousand conformations are imaginable) and the fact that several stereocenters have to be elucidated show that quantum-chemical CD calculations alone cannot determine the absolute configuration of this compound.

At first view, the chromophoric framework of isochromophilone X (**4**) appeared to be comparable to those of **1**–**3**. In fact, calculations of a model of isochromophilone X without the 3,5-dimethyl-1,3-heptadienyl and the 2-hydroxyethyl side chains showed that the transitions in **4** are different from those of the chloroazaphilones **1**–**3**. To obtain reliable information on the absolute configuration of C-7 in **4**, spiking experiments were performed [18]. As described above, **1** is easily converted to **4** by adding 2-aminoethanol. Since this reaction does not change the absolute configuration at C-7, the synthetic isochromophilone X thus prepared had to possess the 7*S*-configuration. The spiking experiment showed that the synthetic and the isolated compound **4** were identical and, consequently possessed the same absolute configuration, 7*S*.

To elucidate the absolute configuration of bartanolide (**6**) the NMR results, especially coupling constants ^3^*J* and NOESY correlation, were analyzed in more depth using quantum-chemical conformation analyses. The NOE between H-3′ and H-9, H-3′ and H-5a, and between H-9 and H-5a turned out to be decisive for the relative configuration of the stereocenters at C-7 and C-9. These correlations are only possible if H-3′ (or the crotonic acid residue), H-9, and H-5a are on the same side of the lactone ring plane, which means that these centers have to be either 7*R*,9*R*- or 7*S*,9*S*-configured. The relative configuration of C-4 was determined using the Karplus equation. The high coupling constant ^3^*J* between H-5a and H-4 of 11.5 Hz showed that the torsional angle between these protons has to be around 180°. Consequently H-4 and H-3′/H-9 have to be located on opposite sides of the lactone-ring plane, leaving only the two enantiomers with the 3*S*,7*R*,9*R*- and 3*R*,7*S*,9*S*-configuration as possible stereoisomers. Starting from the minimum structure found with CAST [19], a conformational analysis of the *S*,*R*,*R*-configuration using B97-D/TZVP gave only twelve structures within an energetic range of 3 kcal/mol. Single-point calculations with SCS-MP2/def2-TZVP and COSMO (methanol) reduced this number of possible conformations to six. Surprisingly, all had the same orientation of the lactone ring, revealing that this part of the molecule is very rigid. The same was true for the *R*,*R*,*R*-configuration, for which we performed the conformational analysis to exclude that a high ^3^*J* value (11.5 Hz) between H-5a and H-4 might occur here because of a possible high flexibility of the lactone ring (see SI). 

**Figure 4:**
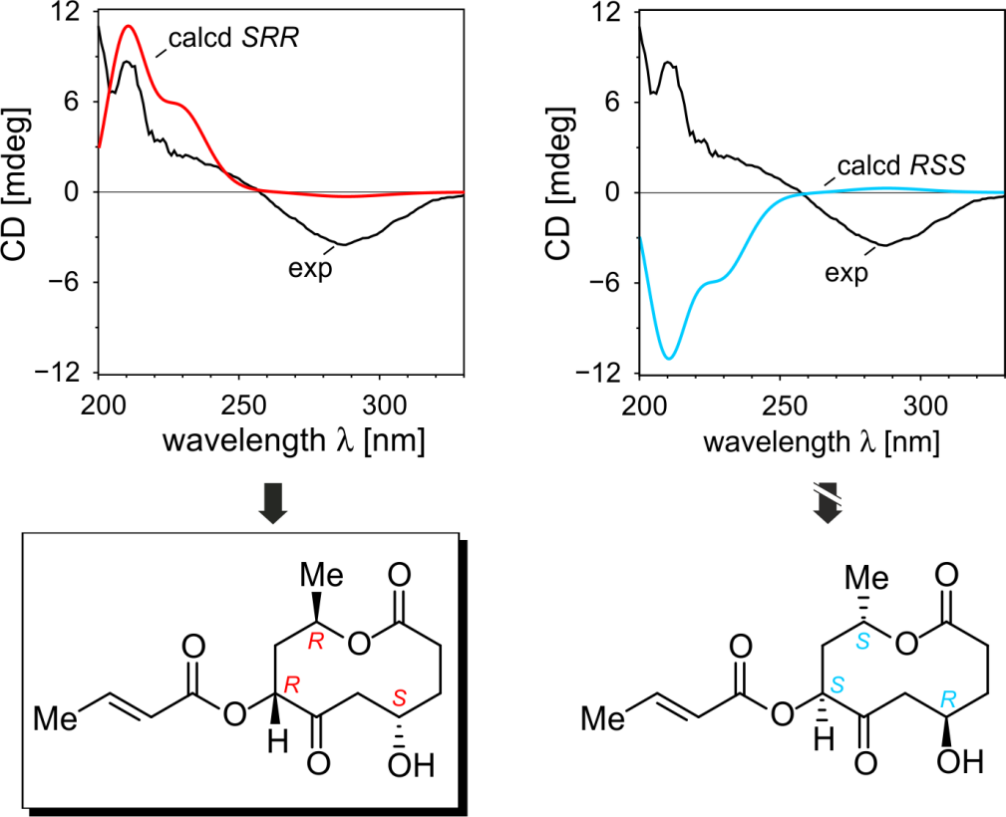
Elucidation of the absolute configuration of bartanolide (**6**) by comparing the CD spectra calculated for *S*,*R*,*R*-**6** and *R*,*S*,*S*-**6** with the experimental spectrum of **6**.

TDB2GP-PLYP/def2-TZVP calculations provided the CD spectra predicted for *S*,*R*,*R*-**6** and *R*,*S*,*S*-**6**. These computed curves were compared with the experimental one [20] of bartanolide (**6**), showing unambiguously that only the CD curve of *S*,*R*,*R*-**6** fits to the measured spectrum (Figure 4).

### 2.2. Inhibitory Activities of the Compounds

Despite their structural similarity, the azaphilones, compounds **1**–**5**, showed remarkable differences in their activity spectra (Table 3). Weak antibacterial activities against *Bacillus subtilis* and *Staphylococcus lentus* were only found in compounds **2** and **5**. Significant inhibition of the yeast *Candida albicans*, the fungi *Trichophyton rubrum and Septoria tritici* was found in compounds **1** and **2**, while **5** revealed specifically weak activity against *T. rubrum*. Inhibition of the enzyme phosphodiesterase 4 (PDE4) was weak by all compounds except **2** exhibiting an IC_50_ value of 2.8 μM. The enzyme acetylcholinesterase was inhibited only by **1** with an IC_50_ value of 2.1 μM. None of the compounds showed cytotoxic activity in our test systems and **6** was inactive in all bioassays.

**Table 3:** IC_50_ values of **1**–**5** in selected bioassays.

	Antibacterial		Antifungal			Enzyme Assays	
	*Bacillus subtilis*	*Staphylococcus lentus*	*Candida albicans*	*Septoria tritici*	*Trichophyton rubrum*	Phosphodiesterase 4	Acetylcholinesterase
helicusin A (**1**)	>100 μM	>100 μM	24.4 μM	7.6 μM (±2.20)	7.23 μM (±1.20)	>10 μM	2.10 μM (±0.36)
deacetylsclerotiorin (**2**)	38.8 μM	43.6 μM	24.0 μM	7.45 μM (±2.05)	2.83 μM (±0.59)	2.79 μM (±0.05)	>50 μM
helicusin E (**3**)	>100 μM	>100 μM	>200 μM	>100 μM	>100 μM	>10 μM	>50 μM
isochromophilone X (**4**)	>100 μM	>100 μM	>100 μM	>100 μM	>80 μM	11.7 μM (±0.80)	not determined
isochromophilone XI (**5**)	55.6 μM	78.4 μM	>100 μM	>100 μM	41.5 μM	8.30 μM (±1.14)	>50 μM
IC_50_ values of the positive controls	chloram-phenicol 1.45 μM (±0.13)	chloram-phenicol 2.13 μM (±0.11)	nystatin 5.80 μM (±2.80)	boscalid 0.53 μM	Clotrimazole 0.2 μM	rolipam 0.75 μM (±0.05)	huperzine <0.1 μM

Arunpanichlert *et al*. [21] observed decreasing activity when a chlorine atom was present in the azaphilone molecule by comparing penicilazaphilone B with (+)-sclerotiorin.

Even though azaphilones have been described [5] with a large variety of bioactivities, such as antimicrobial, antifungal, antiviral, antioxidant, cytotoxic, nematicidal and anti-inflammatory activities, the reported IC_50_ values were mostly rather high. For example, isochromophilone IX has been described with MRSA activity [9], but only with an MIC value of approximately 100 μM.

## 3. Experimental Section

### 3.1. General

The optical rotation was measured on a Perkin-Elmer model 241 polarimeter.

NMR spectra were recorded on a Bruker DRX500 spectrometer (500 and 125 MHz for ^1^H and ^13^C NMR, respectively). The signals of the residual solvent protons and the solvent carbons were used as internal references (δ_H_ 3.35 ppm and δ_C_ 49.3 ppm for MeOH-*d*_4_). High-resolution mass spectra were acquired on a bench top time-of-flight spectrometer (MicrOTOF II, Bruker Daltonics, Germany) by using direct injection with positive electrospray ionization.

Analytical reversed-phase HPLC-DAD/MS analysis was performed using a C18 column (Phenomenex Onyx Monolithic C18, 100 × 3.00 mm) applying an H_2_O/acetonitrile (ACN) gradient with 0.1% formic acid added to both solvents (gradient: 0 min 5% ACN, 4 min 60% ACN, 6 min 100% ACN; flow 2 mL/min) on a Hitachi Elite LaChrom system (DAD-detector: Hitachi L-2450 diode array detector) coupled to an ESI-ion trap detector with positive ionization (Esquire 4000, Bruker Daltonics, Germany).

Semi-preparative HPLC-DAD was performed using a C18 column (Phenomenex Gemini C18 110A Axia, 100 × 21.20 mm) and a Hitachi HPLC system. For preparative fractionation a HPLC-UV system (VWR International LaPrep, Pump P311, Detector P110, autosampler smartline 3900) with a C18 column (Gemini 10u C18, 100A, Axia, 100 × 50.00 mm; gradient: 0 min 15% ACN, 26 min 86% ACN, 27 min 100% ACN; flow: 100 mL/min) was used.

Optical UV/VIS-spectroscopic measurements were performed using a light source (Micropack DT-Mini-2-GS, Ocean Optics Inc., USA) with a wavelength range from 200 to 2000 nm. The cell holder with an absorption cell (100-QS, 10.00 mm, Hellma GmbH & Co. KG, Germany) was connected to the light source and to the detector (USB4000, Ocean Optics, Inc., USA) via optical fibers (QP400-025-SR, Ocean Optics, Inc., USA). CD spectra were recorded on a JASCO J-715 spectropolarimeter using MeOH as a solvent.

### 3.2. Cultivation, Extraction and Substance Characterization

The isolation, cultivation, storage and identification of the strain *Bartalinia robillardoides* LF550 was described by Wiese *et al*. [6].

The strain was revived from Cryobank conserved cultures and grown on WSP30 agar (1.0% glucose·H_2_O, 0.5% peptone from soy meal, 0.3% malt-extract, 0.3% yeast-extract, 3.0% sodium chloride). It was inoculated into 2 L Erlenmeyer flasks with one baffle containing each 1.0 L of a malt-extract medium (1.7% malt-extract, 1.5% sodium chloride). The cultures were shaken at 22 °C and 120 rpm in the dark for 21 days. Thereafter, the 10 L of culture broth were separated into the filtrate and the mycelium. The culture filtrate (approximately 7.0 L) was mixed with 6.0 L ethyl acetate and the organic solvent was separated and concentrated to dryness under reduced pressure. A dark yellow orange powder (1.5 g) was obtained.

The powder was resolved in MeOH. An aliquot of 15 μL of the crude extract was measured by HPLC-DAD/MS. The crude EtOAc extract was subjected to preparative HPLC-UV (Phenomenex Gemini-NX C18 110A, 100 × 50.00 mm; eluents: H_2_O and acetonitrile (ACN); gradient: 0 min 15% ACN, 26 min 86% ACN, 27 min 100% ACN; flow: 100 mL/min; UV detection at 220 nm) and yielded 14 fractions. The substances were eluted as followed: **1** at 22.0 min; **2** at 19.6 min; **3** at 13.3 min; **4** at 16.1 min; **5** at 21.0 min; **6** at 10.5 min.

Helicusin A (**1**): Yellow amorphous solid; 58.5 mg; UV λ_Max_^MeOH^ nm (log ε): 223 (4.33), 267 (sh) (4.01), 286 (4.11), 297 (sh) (4.07), 364 (4.44), 392 (sh) (4.36), 420 (sh) (4.22), 445 (sh) (4.07), 480 (sh) (3.67); ^1^H-NMR (MeOD-*d*_4_, 500 MHz) data were in agreement with those described in the literature [7]; HRESI-MS: *m/z* 475.1533 [M + H^+^] (calcd. for C_25_H_28_^35^ClO_7_: *m/z* 475.1518).

Deacetylsclerotiorin (**2**): (5-Chloro-3-[3,5-dimethyl-1,3-heptadien-1-yl]-7-hydroxy-7-methyl-6*H*-2-benzopyran-6,8(7*H*)-dione): Yellow amorphous solid; 16.3 mg; UV λ_Max_^MeOH^ nm (log ε): 220 (3.99), 262 (3.80), 283 (3.81), 365 (4.05), 385 (sh) (4.02), 420 (sh) (3.88), 444 (sh) (3.69), 480 (sh) (3.25); ^1^H-NMR (600 MHz, CDCl_3_): δ_C_7.94 (1H, s, H-1), 7.09 (1H, d, *J* = 15.6 Hz, H-10), 6.63 (1H, s, H-4), 6.09 (1H, d, *J* = 15.7 Hz, H-9), 5.73 (1H, d, *J* = 10.0 Hz, H-12), 3.42 (1H, s, OH), 2.49 (1H, m, H-13), 1.85 (3H, d, *J* = 1.0 Hz, H-17), 1.59 (3H, s, H-18), 1.44 (1H, m, H-14a), 1.32 (1H, m, H-14b), 1.01 (3H, d, *J* = 6.6 Hz, H-16), 0.86 (3H, t, *J* = 7.5 Hz, H-15); ^13^C NMR (151 MHz, CDCl_3_): δ_C_ 194.0 (C, C-8), 189.7 (C, C-6), 158.6 (C, C-3), 151.6 (CH, C-1), 149.3 (CH, C-12), 146.7 (CH, C-10), 143.4 (C, C-4a), 132.0 (CH, C-11), 118.0 (CCl, C-5), 115.6 (CH, C-9), 115.1 (C, C-8a), 106.0 (CH, C-4), 83.9 (C, C-7), 35.2 (CH, C-13), 30.0 (CH_2_, C-14), 28.8 (CH_3_, C-18), 20.2 (CH_3_, C-16), 12.4 (CH_3_, C-17), 12.0 (CH_3_, C-15). HRESI-MS: *m/z* 349.1205 [M + H^+^] (calcd. for C_19_H_22_^35^ClO_4_: *m/z* 349.1201).

Helicusin E (**3**): yellow amorphous solid; 9.2 mg; UV λ_Max_^MeOH^ nm (log ε): 219 (4.13), 244 (sh) (4.08), 291 (3.99), 300 (sh) (3.98), 359 (4.13), 375 (sh) (4.08), 400 (sh) (3.86), 420 (sh) (3.68), 450 (sh) (3.36), 485 (sh) (2.57); ^1^H-NMR (MeOD-*d*_4_, 500 MHz): δ_H_ 8.23 (1H, s, H-1), 7.01 (1H, d, *J* = 15.7 Hz, H-10), 6.89 (1H, s, H-4), 6.55 (1H, d, *J* = 15.8 Hz, H-9), 5.99 (1H, d, *J* = 1.2 Hz, H-2′), 3.49 (1H, dd, *J* = 3.3, 2.2 Hz, H-12), 3.25 (2H, d, *J* = 0.8 Hz, H-4′), 2.38 (1H, m, H-13), 2.19 (3H, d, *J* = 1.3 Hz, H-6′), 1.58 (3H, s, H-18), 1.49 (1H, m, H-14a), 1.40 (3H, d, *J* = 5.8 Hz, H-16), 1.35 (1H, m, H-14b), 0.95 (3H, t, *J* = 5.4 Hz, H-15), 0.89 (3H, d, *J* = 6.8 Hz, H-17); ^13^C NMR (151 MHz, CDCl_3_): δ_C_ 191.7 (C, C-8), 186.3 (C, C-6), 173.2 (C, C-5′), 164.7 (C, C-1′), 156.6 (C, C-3), 154.3 (C, C-3′), 152.8 (CH, C-1), 145.5 (CH, C-10), 138.5 (C, C-4a), 119.7 (CH, C-9), 117.8 (CH, C-2′), 114.7 (C, C-8a), 111.7 (CCl, C-5), 107.6 (CH, C-4), 84.2 (C, C-7), 79.1 (CH, C-12), 45.5 (CH_2_, C-4′), 35.4 (CH, C-13), 28.63 (CH_2_, C-14), 22.4 (CH_3_, C-18), 19.3 (CH_3_, C-17), 17.7 (CH_3_, C-6′), 13.4 (CH_3_, C-16), 11.9 (CH_3_, C-15); HRESI-MS: *m/z* 509.1573 [M + H^+^] (calcd. for C_25_H_30_^35^ClO_9_: *m/z* 509.1585).

Isochromophilone X (**4**): red amorphous solid; 21.1 mg; [α]_D_^20 ^−138° (c 0.0225, MeOH); UV λ_Max_^MeOH^ nm (log ε): 226 (4.30), 370 (4.25); 1D and 2D ^1^H- and ^13^C-NMR data (MeOD-*d*_4_, 500 MHz and 125 MHz, respectively) see Table 1; HRESI-MS: *m/z* 518.1941 [M + H^+^] (calcd. for C_27_H_33_^35^ClNO_7_: *m/z* 518.1940).

Isochromophilone XI (**5**): yellow amorphous solid, 12.5 mg; UV λ_Max_^MeOH^ nm (log ε): 220 (4.09), 269 (3.75), 395 (4.23); ^1^H-NMR (600 MHz, CDCl_3_). δ_H_ 7.07 (1H, d, *J* = 15.6 Hz, H-10), 6.19 (1H, s, H-4), 6.04 (1H, d, *J* = 15.7 Hz, H-9), 5.94 (1H, s, H-2′), 5.66 (1H, d, *J* = 9.6 Hz, H-12), 4.76 (1H, d, *J* = 12.5 Hz, H-1a), 4.05 (1H, d, *J* = 12.5 Hz, H-1b), 3.18 (2H, s, H-4′), 2.46 (1H, m, H-13), 2.15 (3H, s, H-6′), 1.85 (3H, s, H-17), 1.82 (3H, s, H-18), 1.41 (1H, m, H-14a), 1.31 (1H, m, H-14b), 0.99 (3H, d, *J* = 6.6 Hz, H-16), 0.86 (3H, t, *J* = 7.4 Hz, H-15); ^13^C NMR (151 MHz, CDCl_3_) δ_C_ 197.4 (C, C-8), 185.7 (C, C-6), 173.6 (C, C-5′), 164.2 (C, C-1′), 162.4 (C, C-3), 154.1 (C, C-3′), 148.1 (CH, C-12), 143.1 (C, C-4a), 142.9 (CH, C-10), 132.3 (C, C-11), 121.2 (CCl, C-5), 118.3 (CH, C-2′), 117.8 (CH, C-9), 100.4 (CH, C-4), 83.2 (C, C-7), 70.1 (CH_2_, C-1), 68.0 (C, C-8a), 45.5 (CH_2_, C-4′), 35.1 (CH, C-13), 30.1 (CH_2_, C-14), 23.6 (CH_3_, C-18), 20.3 (CH_3_, C-16), 19.3 (CH_3_, C-6′), 12.4 (CH_3_, C-17), 12.0 (CH_3_, C-15); HRESI-MS: *m/z* 493.1625 [M + H^+^] (calcd. for C_25_H_30_^35^ClO_8_: *m/z* 493.1624).

Bartanolide (**6**): colorless amorphous solid; 3.6 mg; [α]_D_^20 ^−36° (c 0.210, MeOH); UV λ_Max_^MeOH^ nm (log ε): 220 (4.04); 1D and 2D ^1^H- and ^13^C-NMR data (MeOD-*d*_4_, 500 MHz and 125 MHz, respectively) see Table 2; HRESI-MS: *m/z* 285.1338 [M + H^+^] (calcd. for C_14_H_21_O_6_: *m/z* 285.1333).

### 3.3. Bioassays

*Bacillus subtilis*, *Staphylococcus lentus*, *Xanthomonas campestris* and *Candida albicans*. The antimicrobial assays were performed as reported by Schulz *et al*. [22]. 

*Septoria tritici*. The substances were dissolved in DMSO to a concentration of 10 mM and transferred into a 96-well microtiter plate. For screening purposes, the compounds were tested in a final concentration of 100 μM. The fungal strain *S. tritici* was cultured on MYG medium (1.0% malt extract, 0.4% yeast extract, 0.4% glucose·H_2_O, pH = 5.6) for four days, subsequently the culture was diluted to an optical density (600 nm) of 0.03 and added to the microtiter plate. After incubation for 48 h at 20 °C, the optical density was measured. The resulting values were compared to a positive (0.53 μM boscalid) and a negative control (no compound) on the same plate.

*Trichophyton rubrum.* The substances were dissolved in DMSO to a concentration of 10 mM and transferred into a 96-well microtiter plate. For screening purposes, the compounds were tested in a final concentration of 100 μM. For inoculation 5 × 10^4^ spores mL^−1^ from the fungal strain *T. rubrum* diluted in Sabouraud medium (1.0% peptone, 2.0% glucose·H_2_O, pH = 5.6) were added to the microtiter plate. After incubation for 72 h at 28 °C the optical density was measured. The resulting values were compared with a positive (0.5 μM clotrimazole) and a negative control (no compound) on the same plate.

*Phytophtora infestans*. The substances were dissolved in DMSO to a concentration of 10 mM and transferred into a 96-well microtiter plate. For screening purposes, the compounds were tested in a final concentration of 100 μM. For inoculation 10^4^ spores mL^−1^ from *P. infestans* diluted in pea medium (filtrate of 150 g cooked peas per liter pure water added with 5.0 g glucose·H_2_O and 0.1 mg thiamine, pH = 6.5) were added to the microtiter plate. After incubation for 72 h at 20 °C the optical density was measured. The resulting values were compared with a positive (0.5 μM clotrimazole) and a negative control (no compound) on the same plate.

Cytotoxicity assays. The sensitivity of the cell lines KIF5 and HepG2 to the isolated compounds was evaluated by monitoring the metabolic activity using the *CellTiterBlue* Cell Viability Assay (Promega, Mannheim, Germany). The human fibroblast cell line (KIF5) was provided by the Institute for Experimental Tumor Research, Section Molecular Oncology, Kiel, Germany, and the human hepatocellular carcinoma cell line (HepG2) was obtained from the German Collection of Microorganism and Cell Cultures (DSMZ, Braunschweig, Germany). The cytotoxicity assays were performed as described by Schulz *et al*. [22], apart from the cell concentration, which were 5000 KIF5 cells and 10,000 HepG2 cells per vial.

Enzyme inhibition assays PDE4 and AChE assays were performed as described by Schulz *et al*. [22] and Kim *et al*. [23], respectively.

### 3.4. Computational Details

The conformational analyses of the two diastereomers of **6** were started with CAST [19] using the OPLS_AA forcefield [24] with subsequent PM7 optimizations using MOPAC2012 [25]. From the thus found lowest conformations a more in-depth analysis was performed using B97-D/TZVP [26,27] within Gaussian09 [28]. To get more reliable energies SCS-MP2/def2-TZVP [29,30] in combination with COSMO (methanol) [31] single-point calculations were done. UV and CD spectra of the found lowest-energy conformations were calculated with TDB2GP-PLYP/def2-TZVP [30,32] and COSMO (methanol). Both SCS-MP2 and TD calculations were performed using the chain-of-spheres approximation [33,34] with ORCA [35]. Boltzmann-weighting, overlay with Gaussians (σ = 0.3 eV), UV shift (12 nm), and comparisons with the experiment were carried out with SpecDis [36].

## 4. Conclusions

Three new chloroazaphilones (isochromophilone X, isochromophilone XI and helicusin E) and one new pentaketide (bartanolide) along with two known metabolites were discovered in *Bartalinia robillardoides* LF550, isolated from a marine sponge. Dong *et al*. [37] postulated that azaphilones were effective pest-managing agents that might offer alternatives to some synthetic agents. Bell *et al*. [38] reported that the selective reactivity and fluorescent behavior of the azaphilone epicocconone could be exploited to enable real-time imaging of living cells and the study of organelle movements. Following these suggestions, our three new chloroazaphilones may as well be used in these or similar applications. Their bioactivity spectra give first hints at possible applications in the treatment of specific diseases.

## Supplementary Files

Supplementary File 1 Supplementary Information (PDF, 2042 KB)
