# Supplementary material for: Helicusin E, Isochromophilone X and Isochromophilone XI: New Chloroazaphilones Produced by the Fungus Bartalinia robillardoides Strain LF550
Source: Mar Drugs. 2013 Mar 12;11(3):800–16. doi: 10.3390/md11030800 (PMC3705371; doi:10.3390/md11030800)

Supplementary Information

Figure S1. ^{1}H-NMR of compound helicusin A (**1**) in MeOD-d_4. 2
Figure S2. ^{1}H-NMR of compound deacetylsclerotiorin (**2**) in $CDCl_3$. 2
Figure S3. HPLC-CD of compound deacetylsclerotiorin (**2**). 3
Figure S4. ^{1}H-NMR of compound deacetylsclerotiorin (**2**) in CDCl3 showing traces of the Z isomer. 3
Figure S5. ^{1}H-NMR of compound helicusin E (**3**) in MeOD-d_4. 4
Figure S6. ^{1}H-NMR of compound isochromophilone X (**4**) in MeOD-d_4. 4
Figure S7. ^{13}C-NMR of compound isochromophilone X (**4**) in MeOD-d_4. 5
Figure S8. COSY of compound isochromophilone X (**4**) in MeOD-d_4. 5
Figure S9. HSQC of compound isochromophilone X (**4**) in MeOD-d_4. 6
Figure S10. HMBC of compound isochromophilone X (**4**) in MeOD-d_4. 6
Figure S11. NOESY of compound isochromophilone X (**4**) in MeOD-d_4. 7
Figure S12. HPLC-CD of compound isochromophilone X (**4**). 8
Figure S13. ^{1}H-NMR of compound isochromophilone XI (**5**) in MeOD-d_4. 9
Figure S14. ^{1}H-NMR of compound bartanolide (**6**) in MeOD-d_4. 9
Figure S15. COSY of compound bartanolide (**6**) in MeOD-d_4. 10
Figure S16. ^{13}C-NMR of compound bartanolide (**6**) in MeOD-d_4. 10
Figure S17. 135-DEPT of compound bartanolide (**6**) in MeOD-d_4. 11
Figure S18. HSQC of compound bartanolide (**6**) in MeOD-d_4. 11
Figure S19. HMBC of compound bartanolide (**6**) in MeOD-d_4. 12
Figure S20. Minimum Conformers of *S*,*R*,*R*-6 and *R*,*R*,*R*-6. 12
Figure S21. NOESY of compound bartanolide (**6**) in MeOD-d_4. 13

Figure S1. ^{1}H-NMR of compound helicusin A (**1**) in MeOD-d_4.

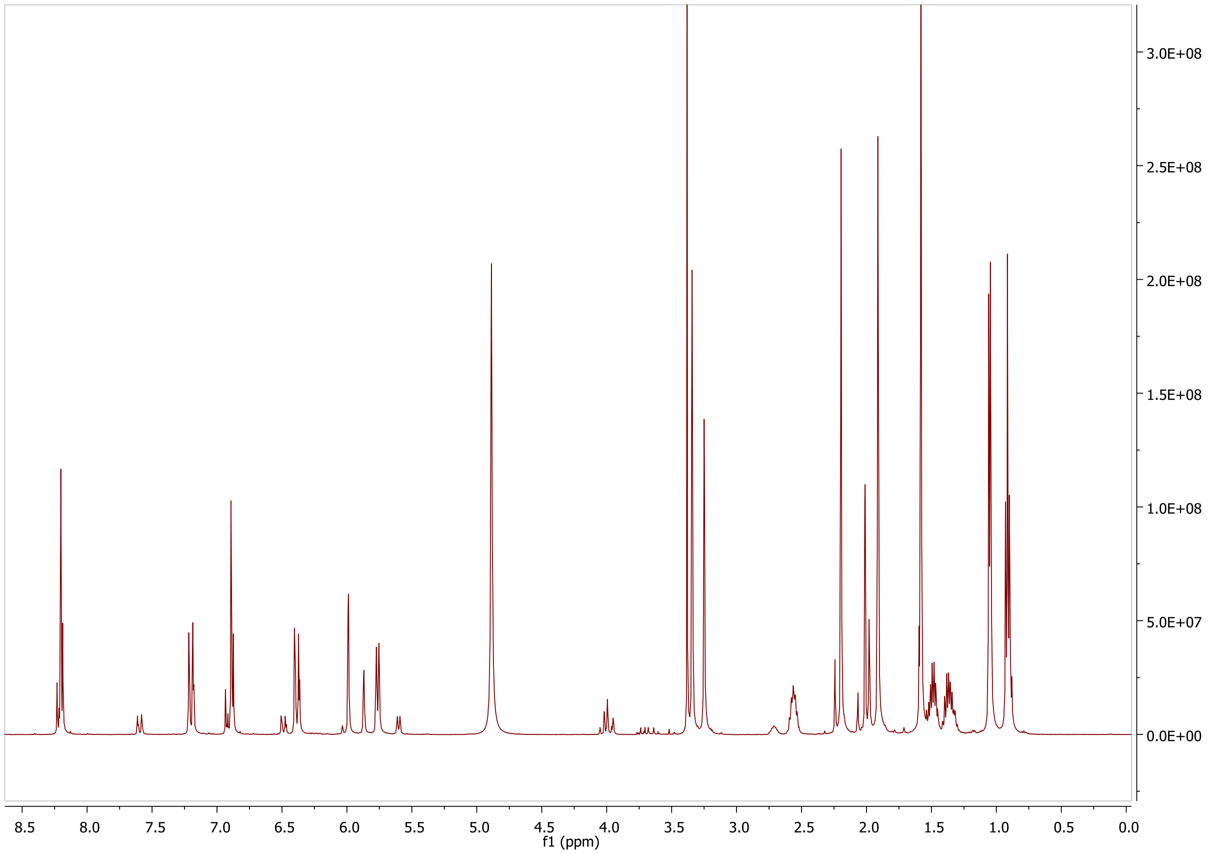

Figure S2. ^{1}H-NMR of compound deacetylsclerotiorin (**2**) in $CDCl_3$.

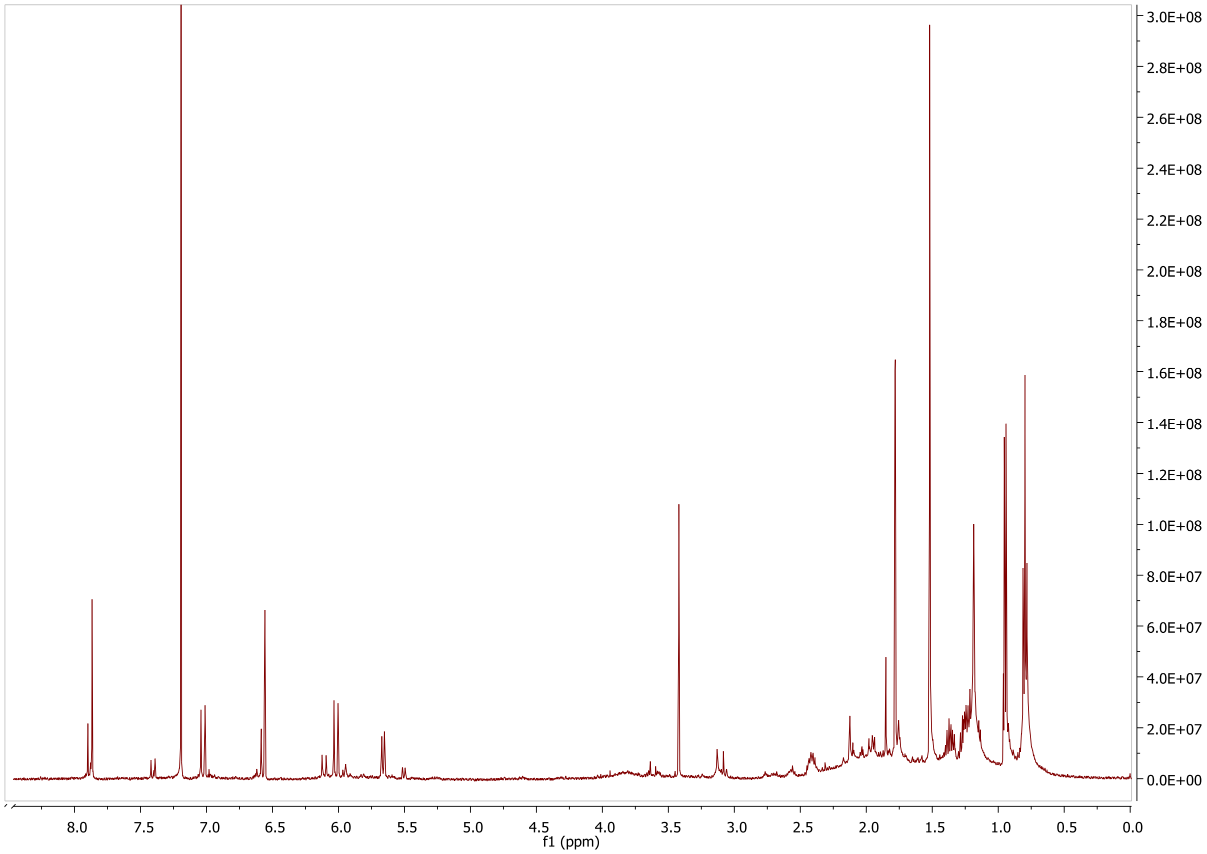

Figure S3. HPLC-CD of compound deacetylsclerotiorin (**2**).

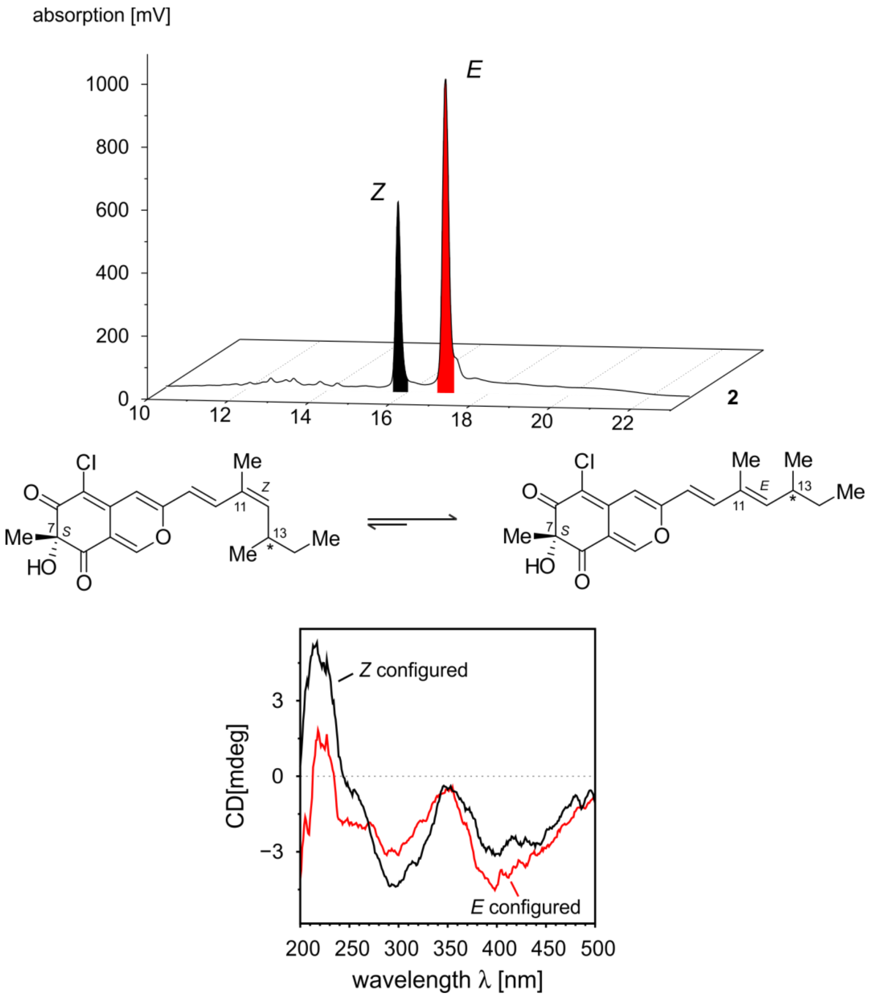

Figure S4. ^{1}H-NMR of compound deacetylsclerotiorin (**2**) in CDCl3 showing traces of the *Z* isomer.

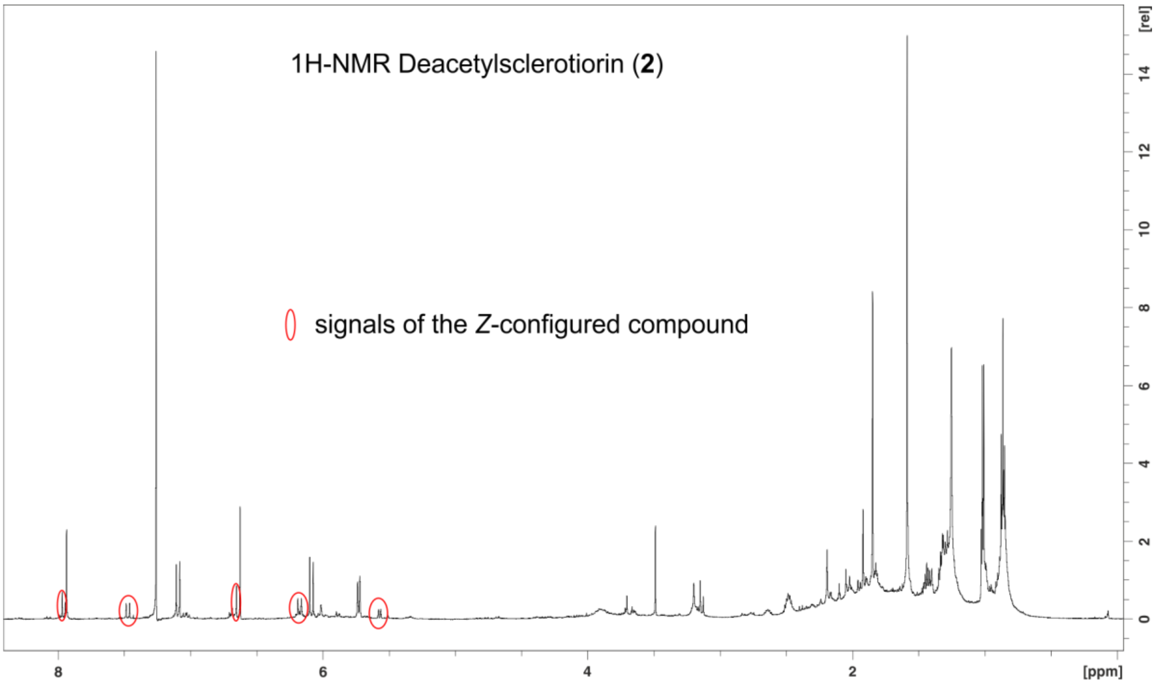

Figure S5. ^{1}H-NMR of compound helicusin E (**3**) in MeOD-d_4.

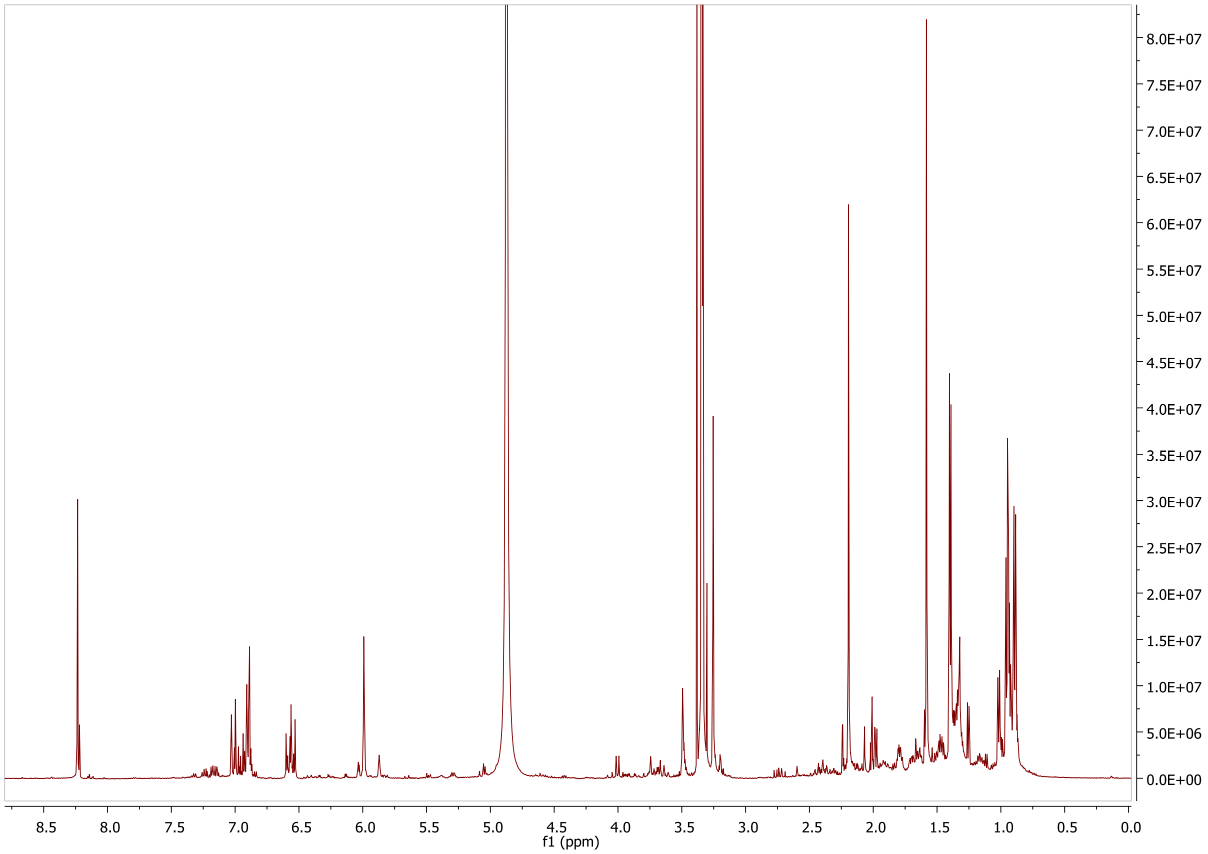

Figure S6. ^{1}H-NMR of compound isochromophilone X (**4**) in MeOD-d_4.

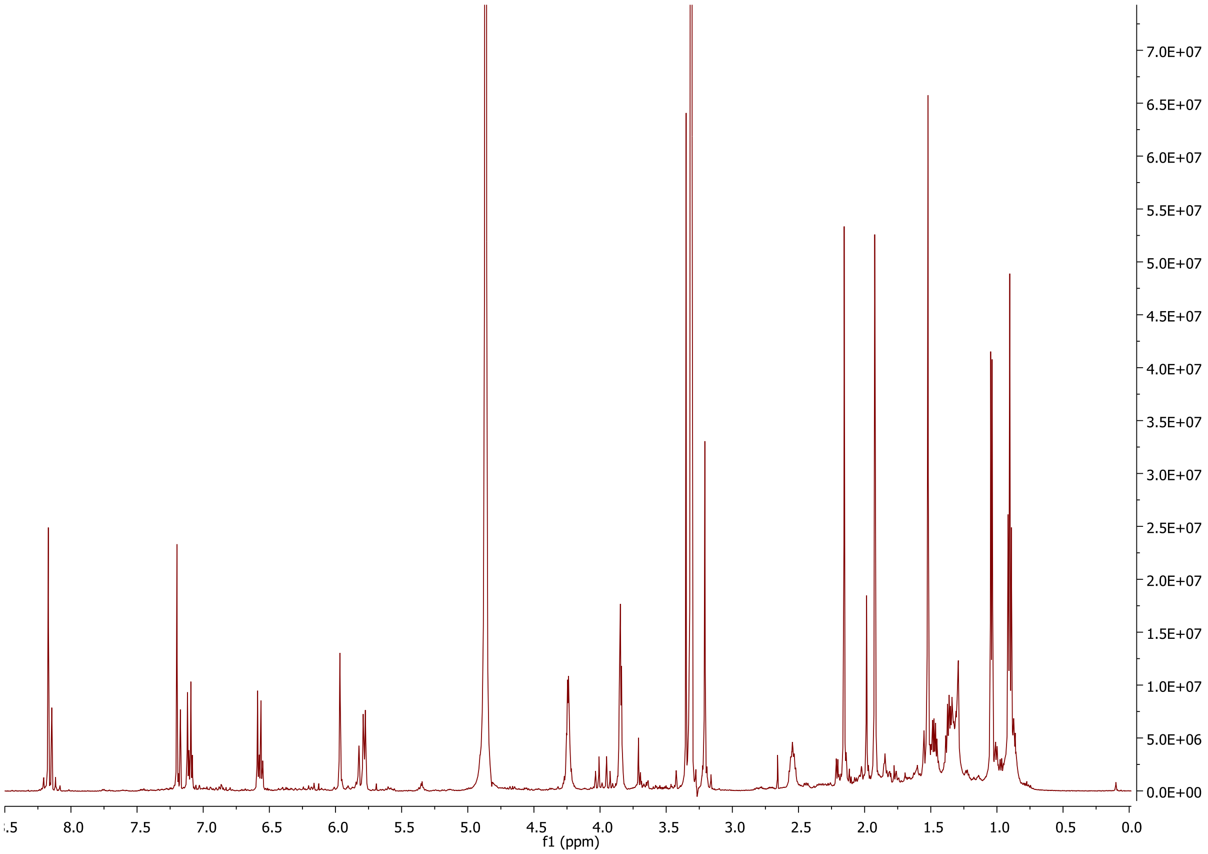

Figure S7. ^{13}C-NMR of compound isochromophilone X (**4**) in MeOD-d_4.

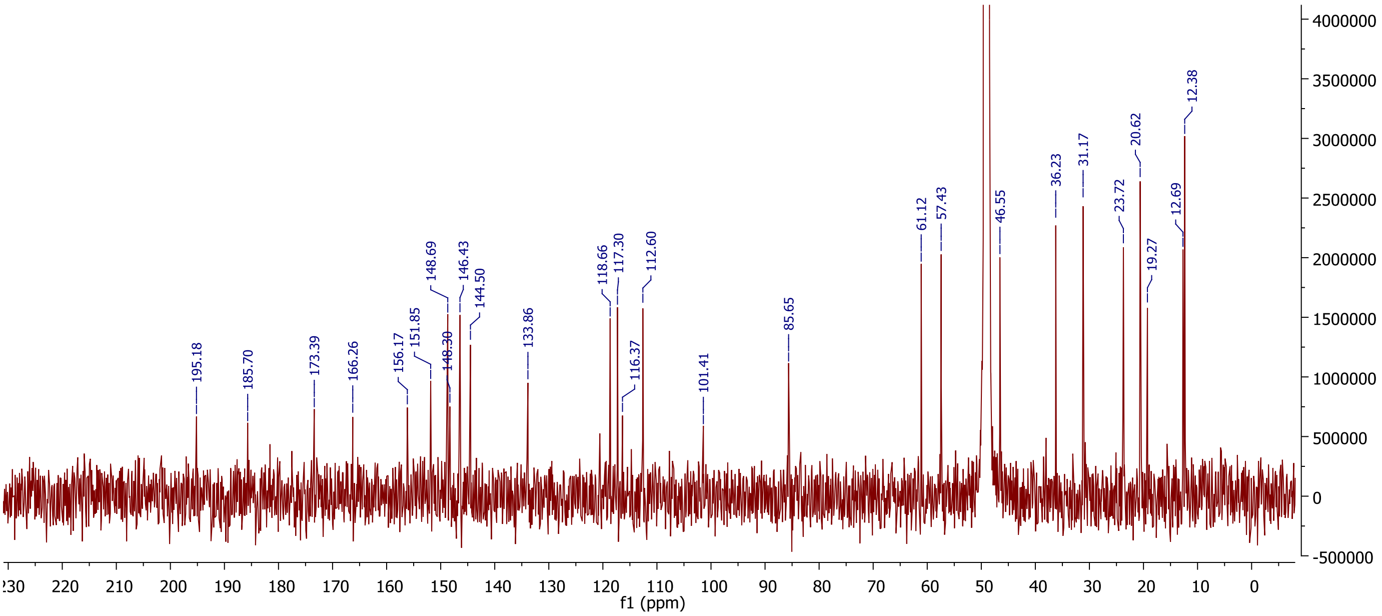

Figure S8. COSY of compound isochromophilone X (**4**) in MeOD-d_4.

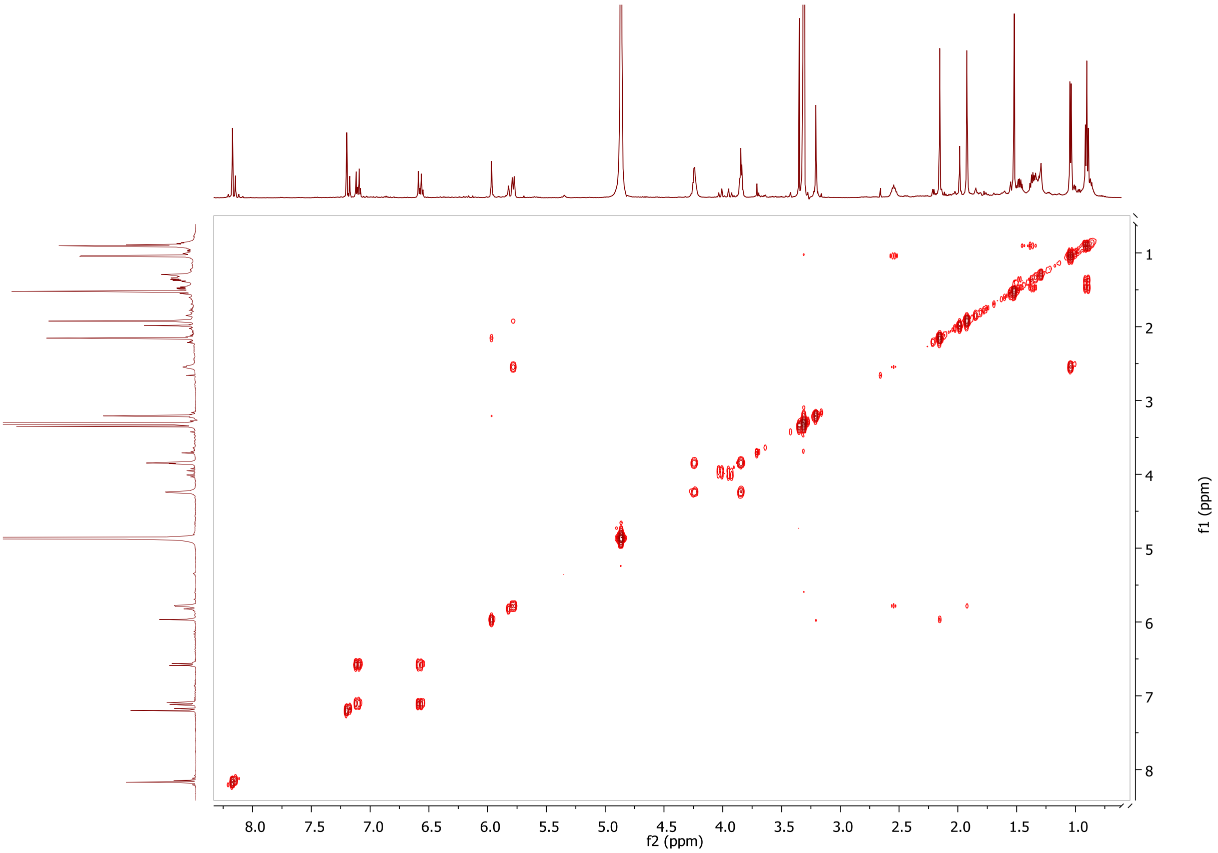

Figure S9. HSQC of compound isochromophilone X (**4**) in MeOD-d_4.

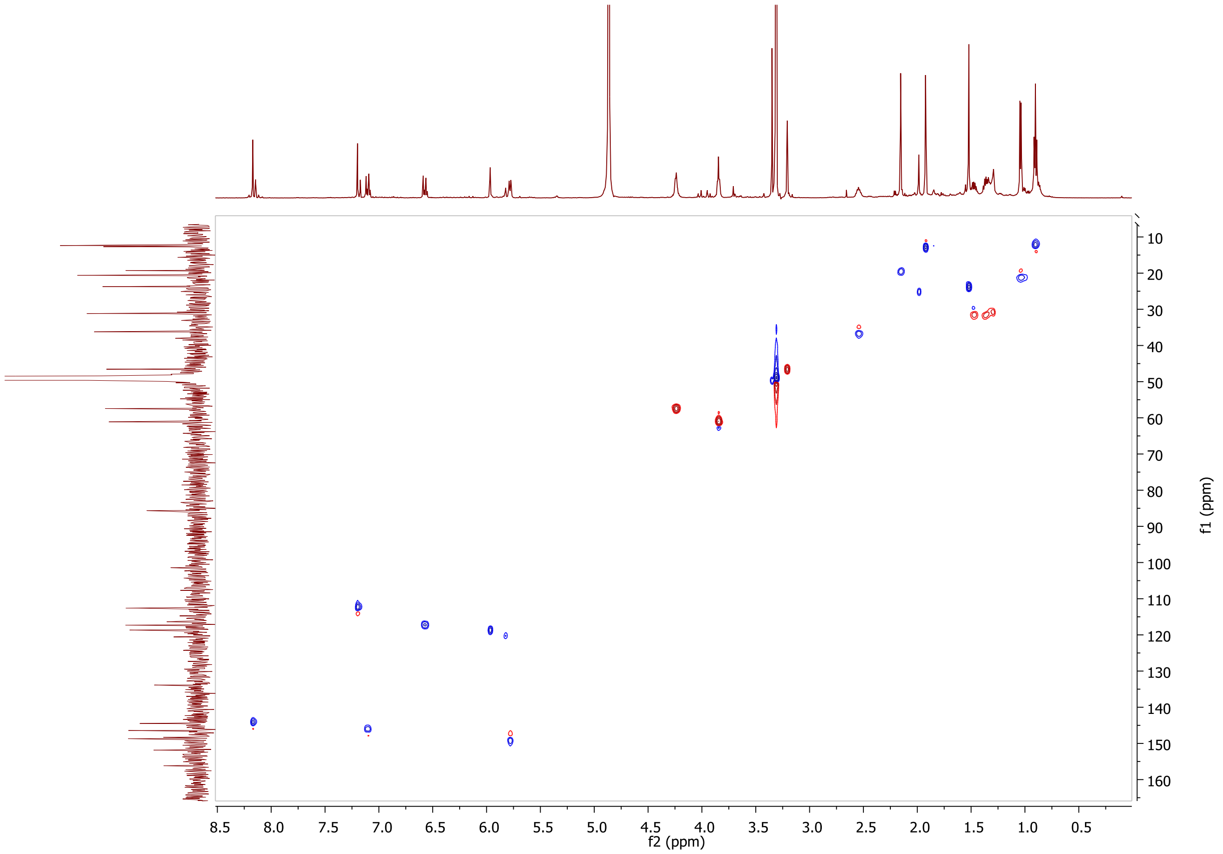

Figure S10. HMBC of compound isochromophilone X (**4**) in MeOD-d_4.

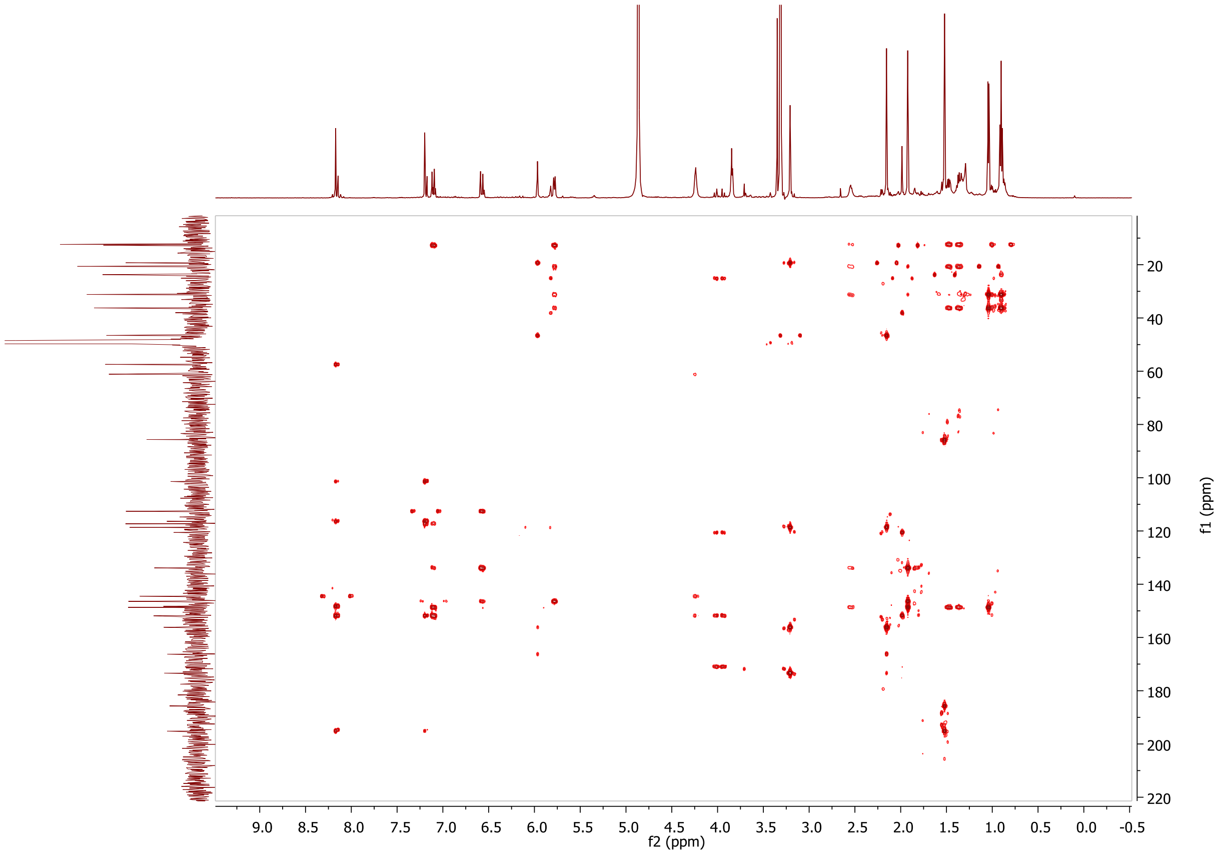

Figure S11. NOESY of compound isochromophilone X (**4**) in MeOD-d_4.

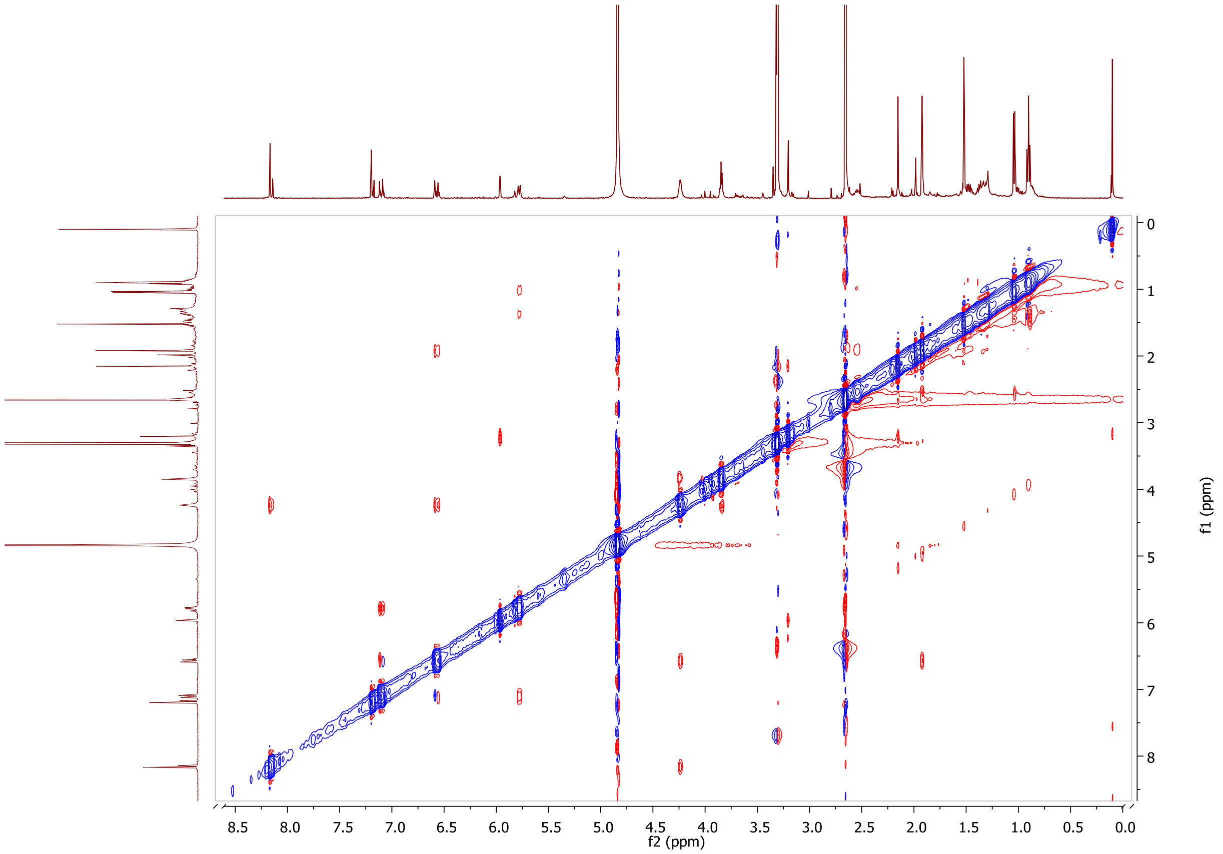

Figure S12. HPLC-CD of compound isochromophilone X (**4**).

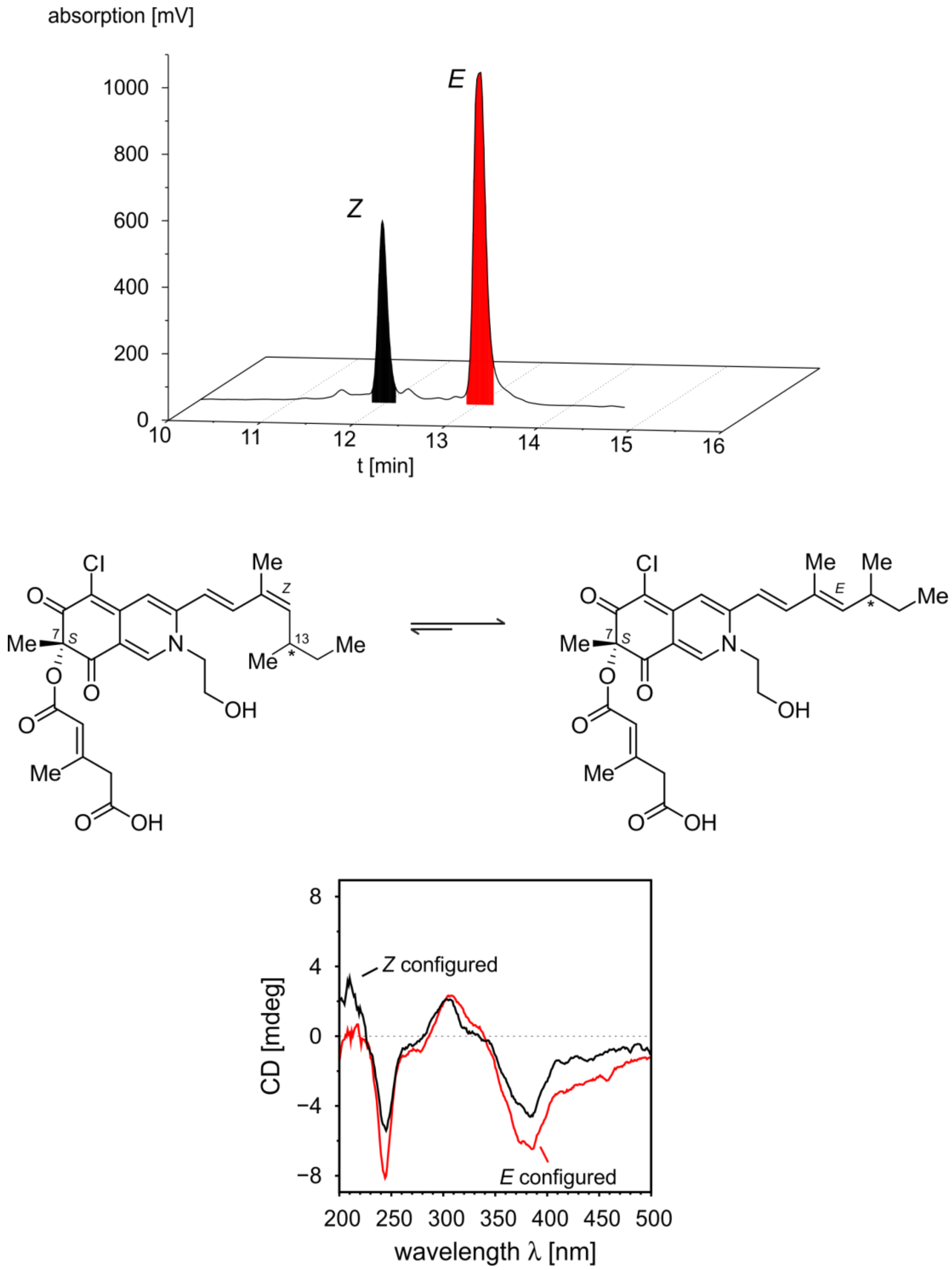

Figure S13. ^{1}H-NMR of compound isochromophilone XI (**5**) in MeOD-d_4.

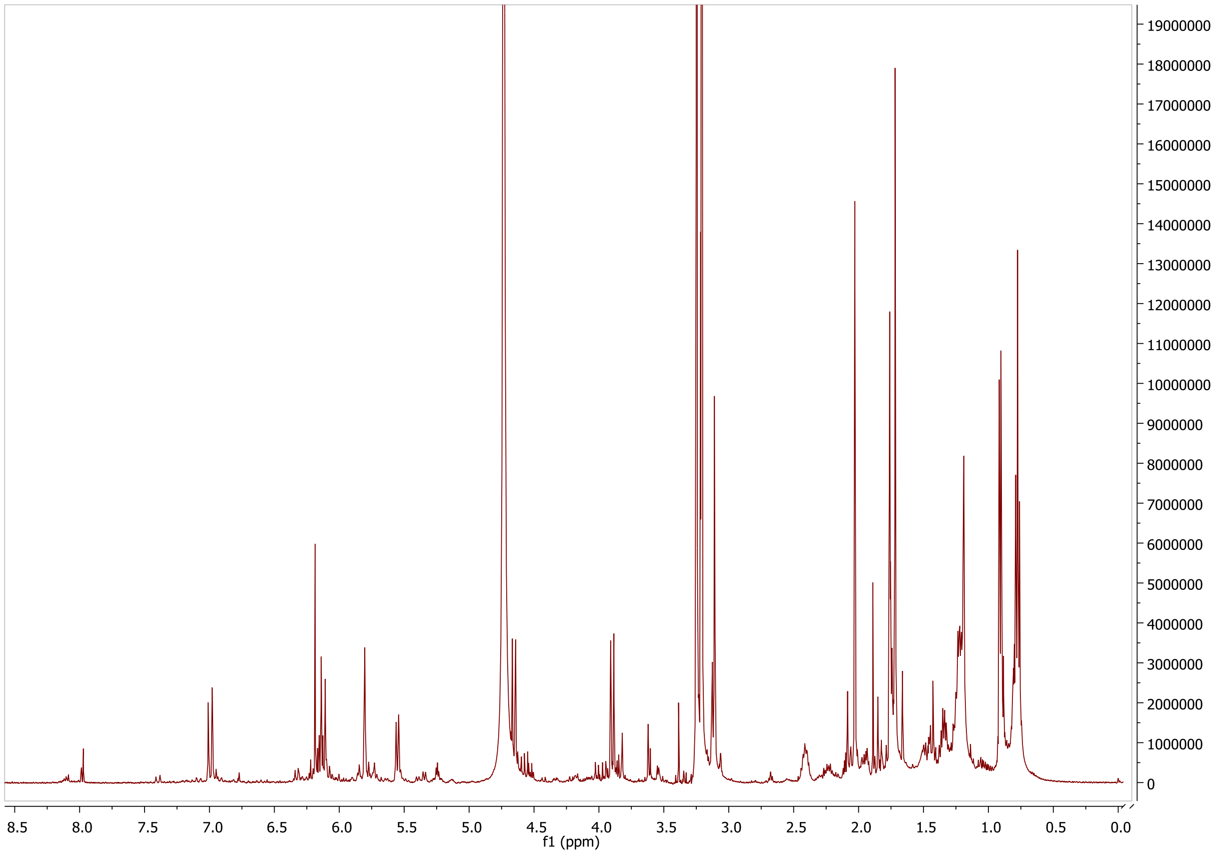

Figure S14. ^{1}H-NMR of compound bartanolide (**6**) in MeOD-d_4.

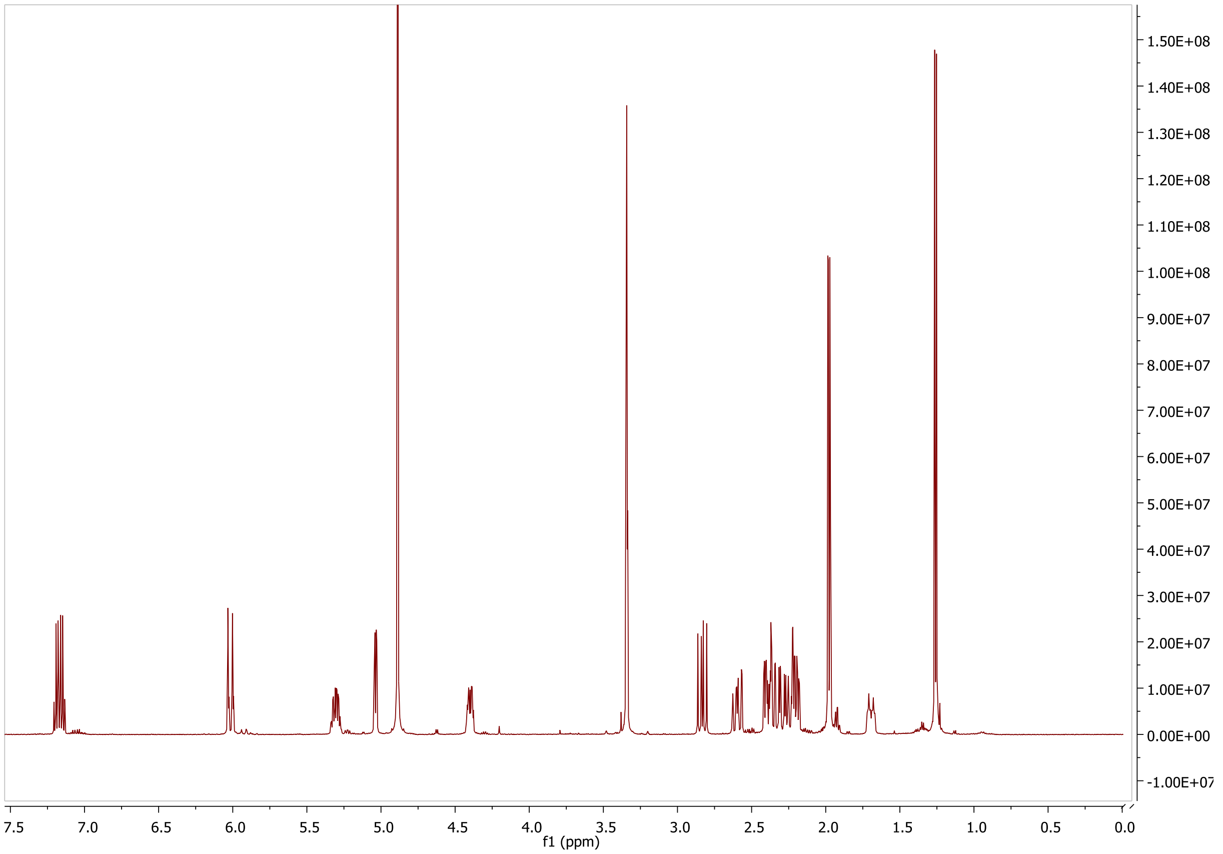

Figure S15. COSY of compound bartanolide (**6**) in MeOD-d_4.

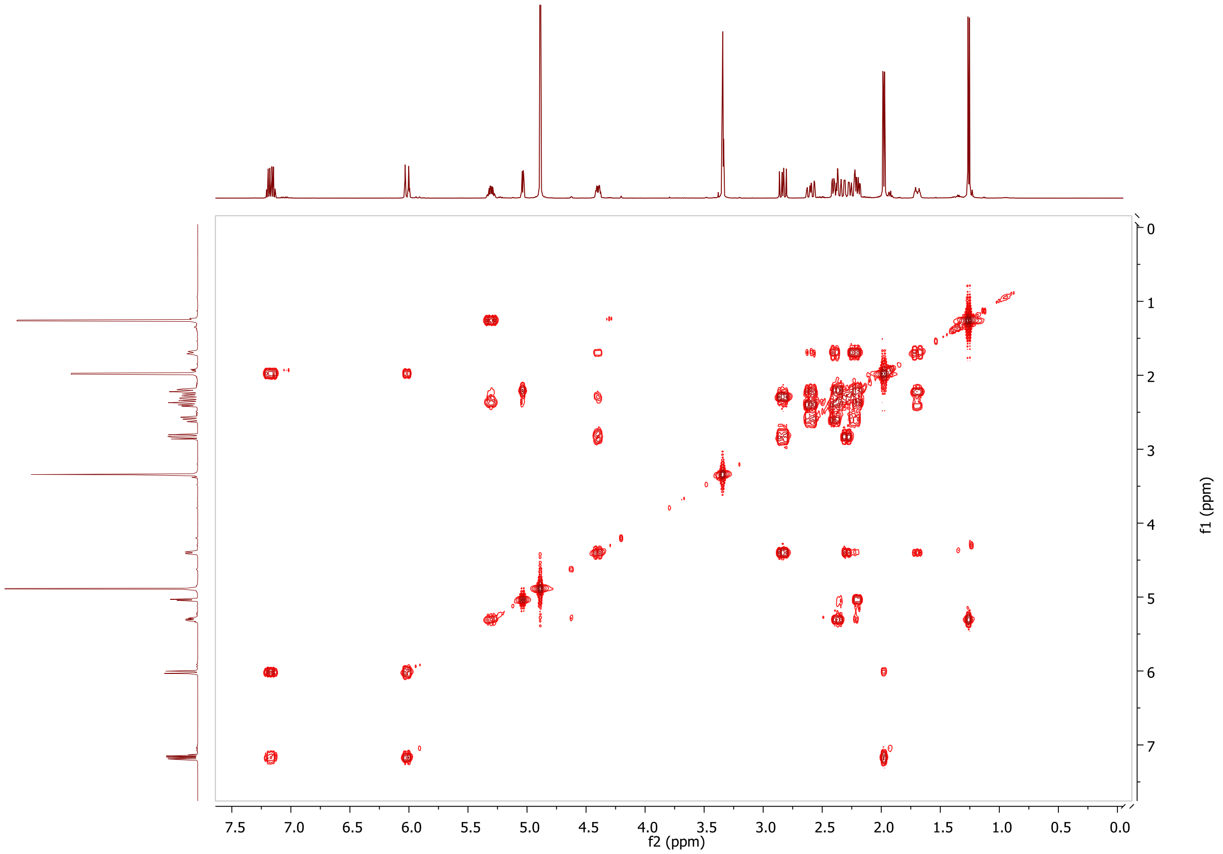

Figure S16. ^{13}C-NMR of compound bartanolide (**6**) in MeOD-d_4.

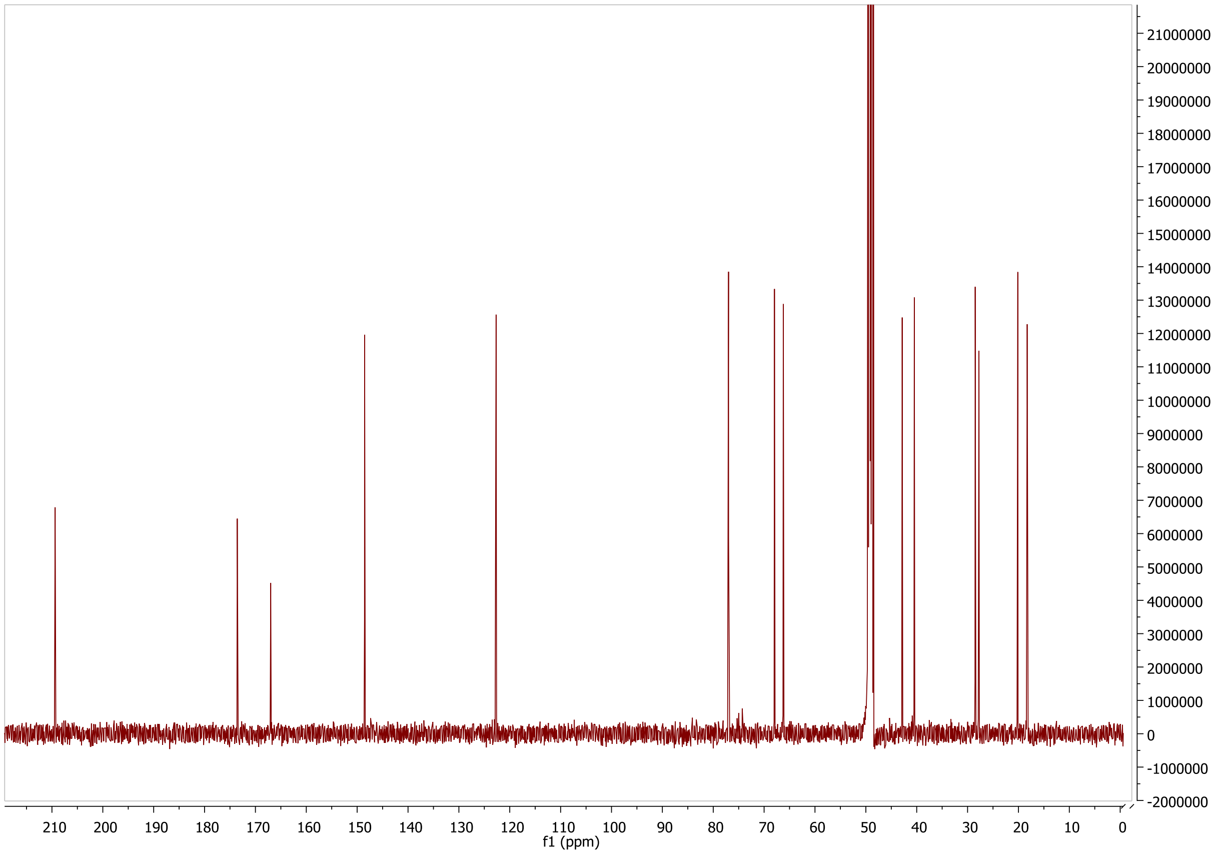

Figure S17. 135-DEPT of compound bartanolide (**6**) in MeOD-d_4.

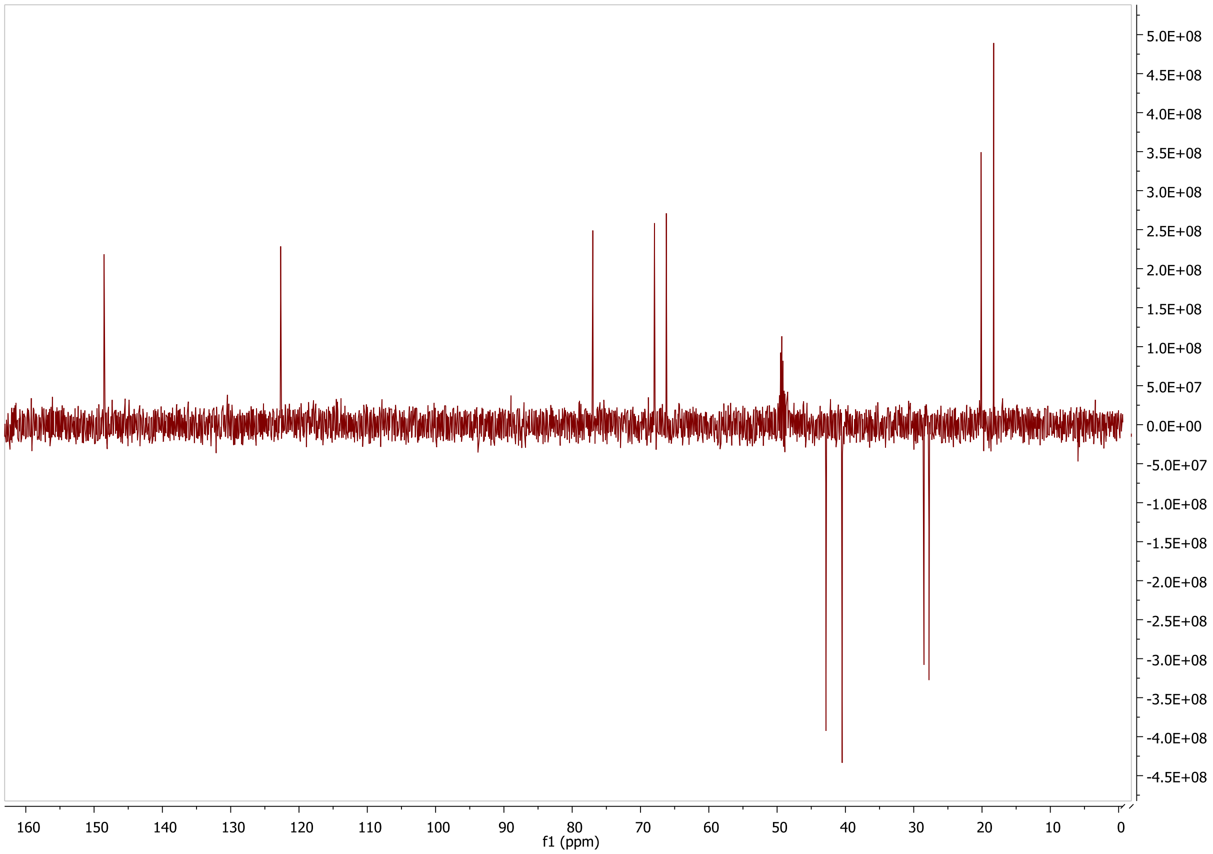

Figure S18. HSQC of compound bartanolide (**6**) in MeOD-d_4.

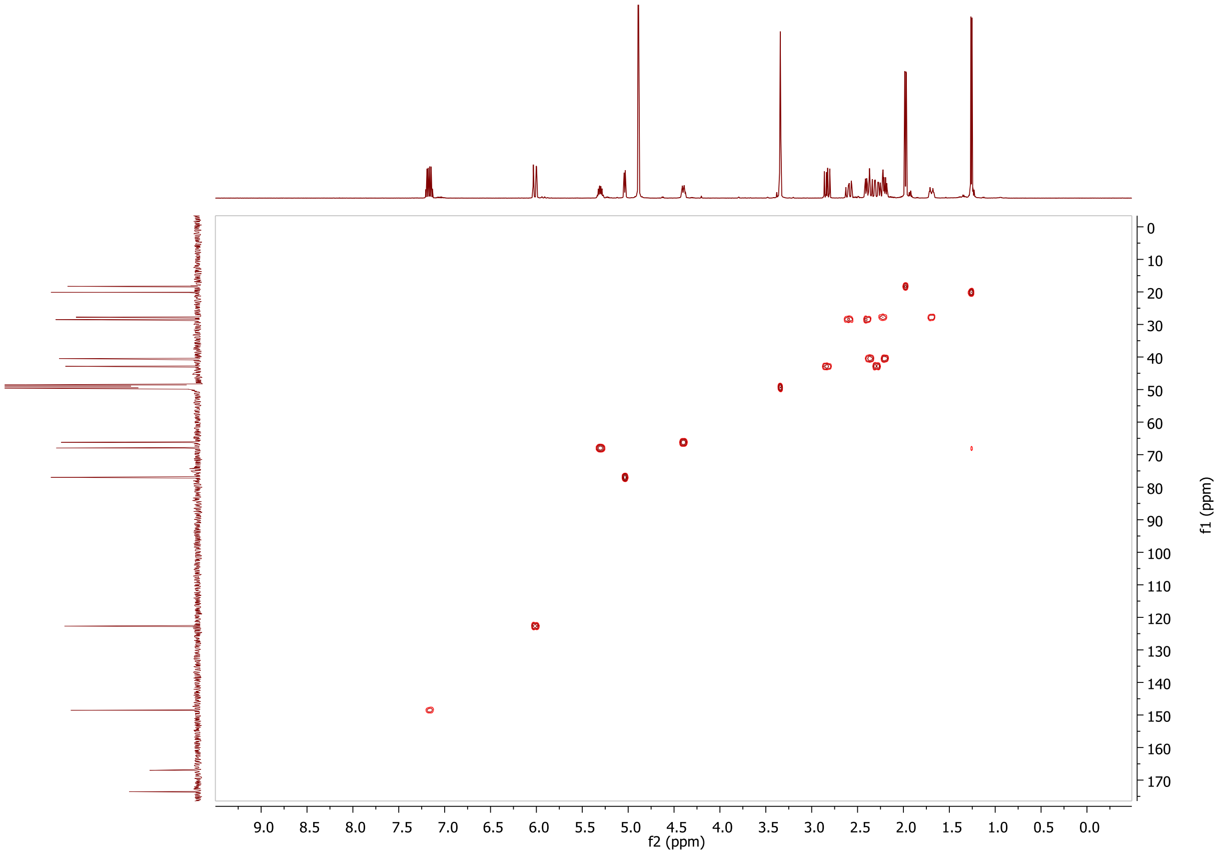

Figure S19. HMBC of compound bartanolide (**6**) in MeOD-d_4.

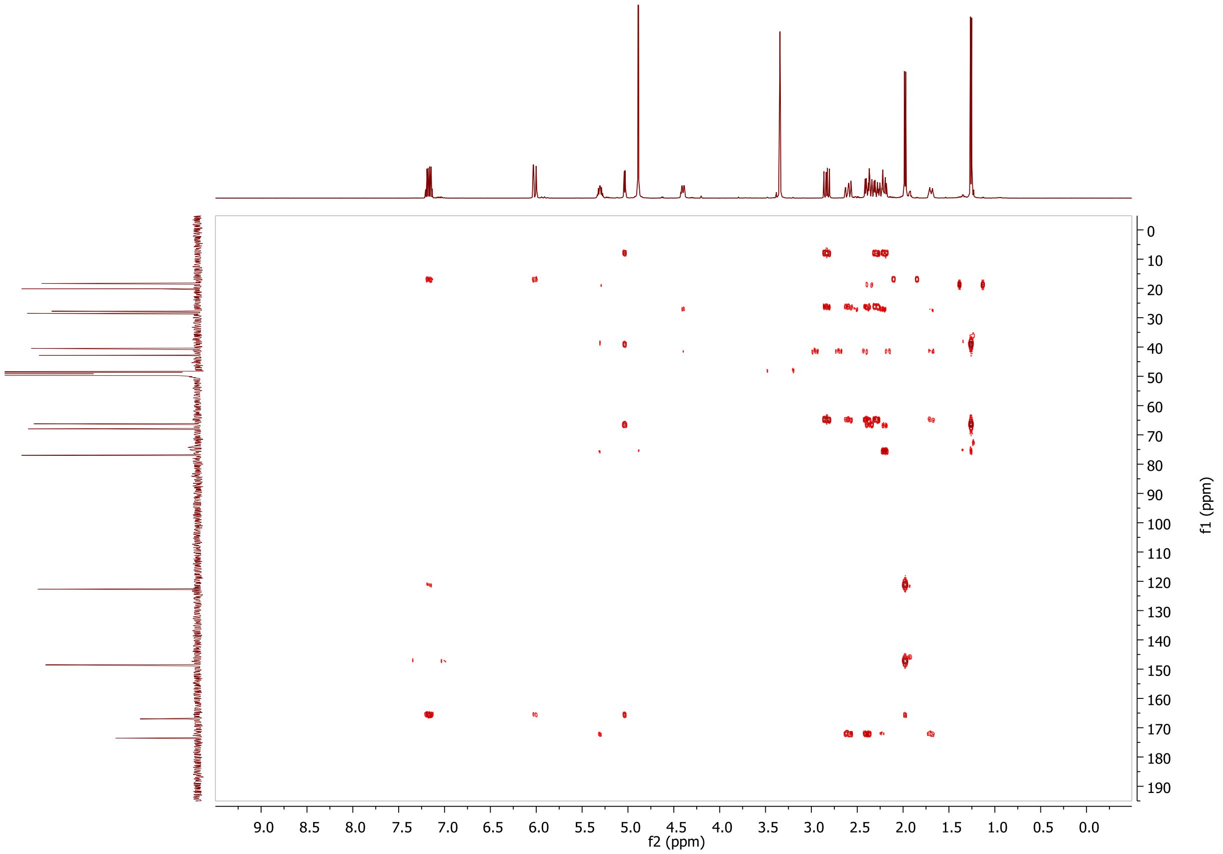

Figure S20. Minimum Conformers of *S*,*R*,*R*-6 and *R*,*R*,*R*-6.

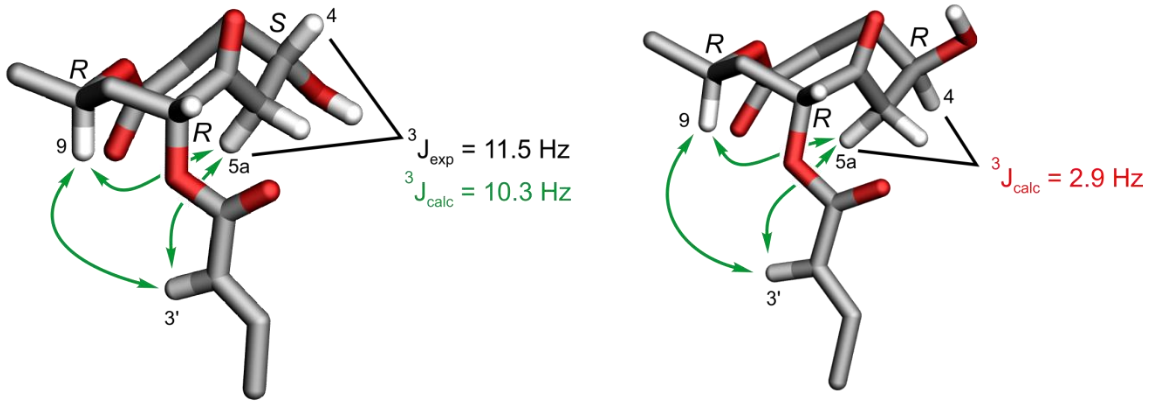

The found weak NOE between H-9 and H-5a and between H-9 and H-3′ as well as the NOE between H-3′ and H-5a were possible in the *S*,*R*,*R*- and the *R*,*R*,*R*-configurations (and in their enantiomers) of **6**. Conformational analyses with the B97D/TZVP method gave the minimum energy conformers shown above. Using the Karplus equation and NMR calculations (B3LYP/6-31G**//B97D/TZVP, CPCM, solvent = methanol) of the ^3J coupling between H-5a and H-4 it became clear that only the *S*,*R*,*R* configuration and its enantiomer fit to the experimental results.

Figure S21. NOESY of compound bartanolide (**6**) in MeOD-d_4.

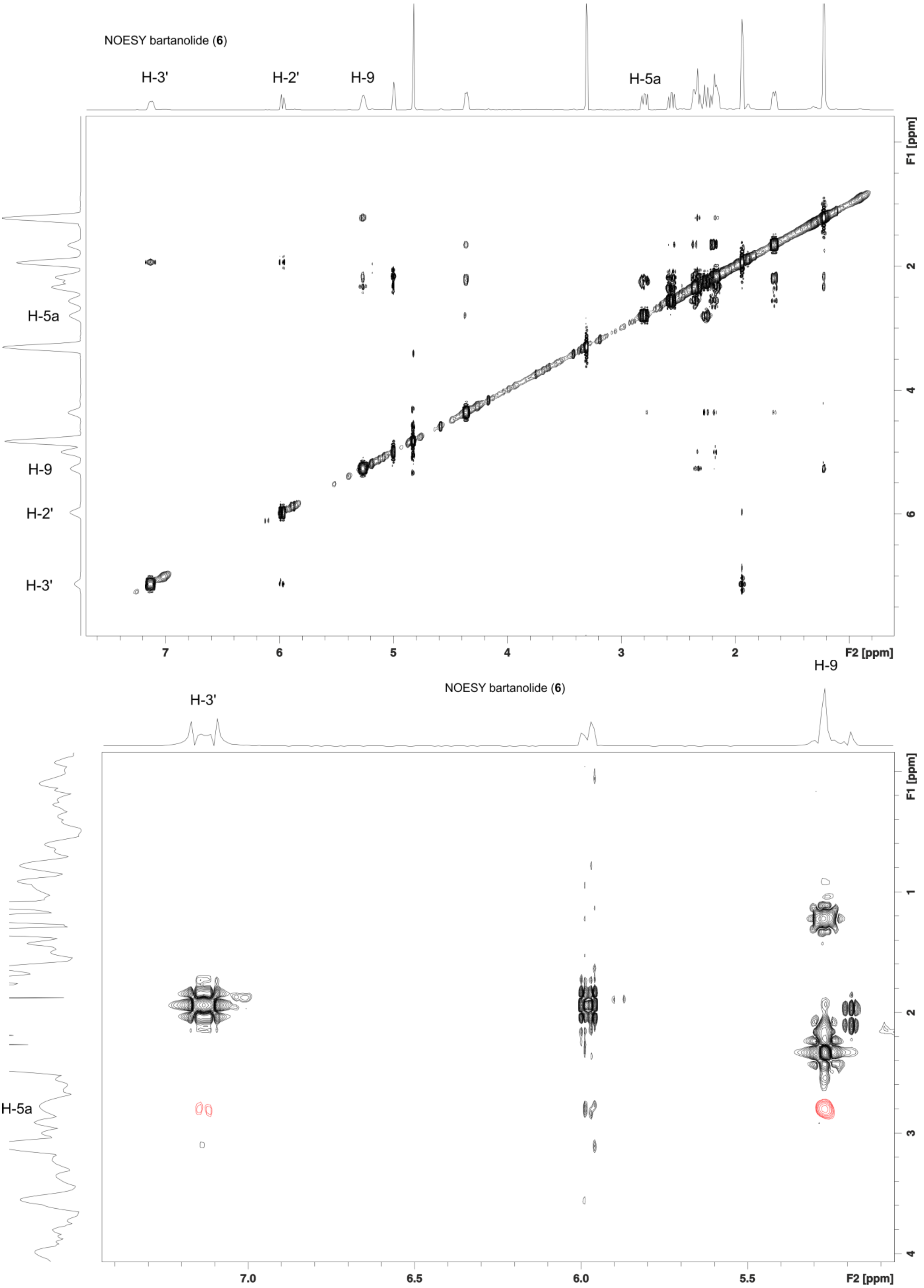

Figure S21. *Cont.*

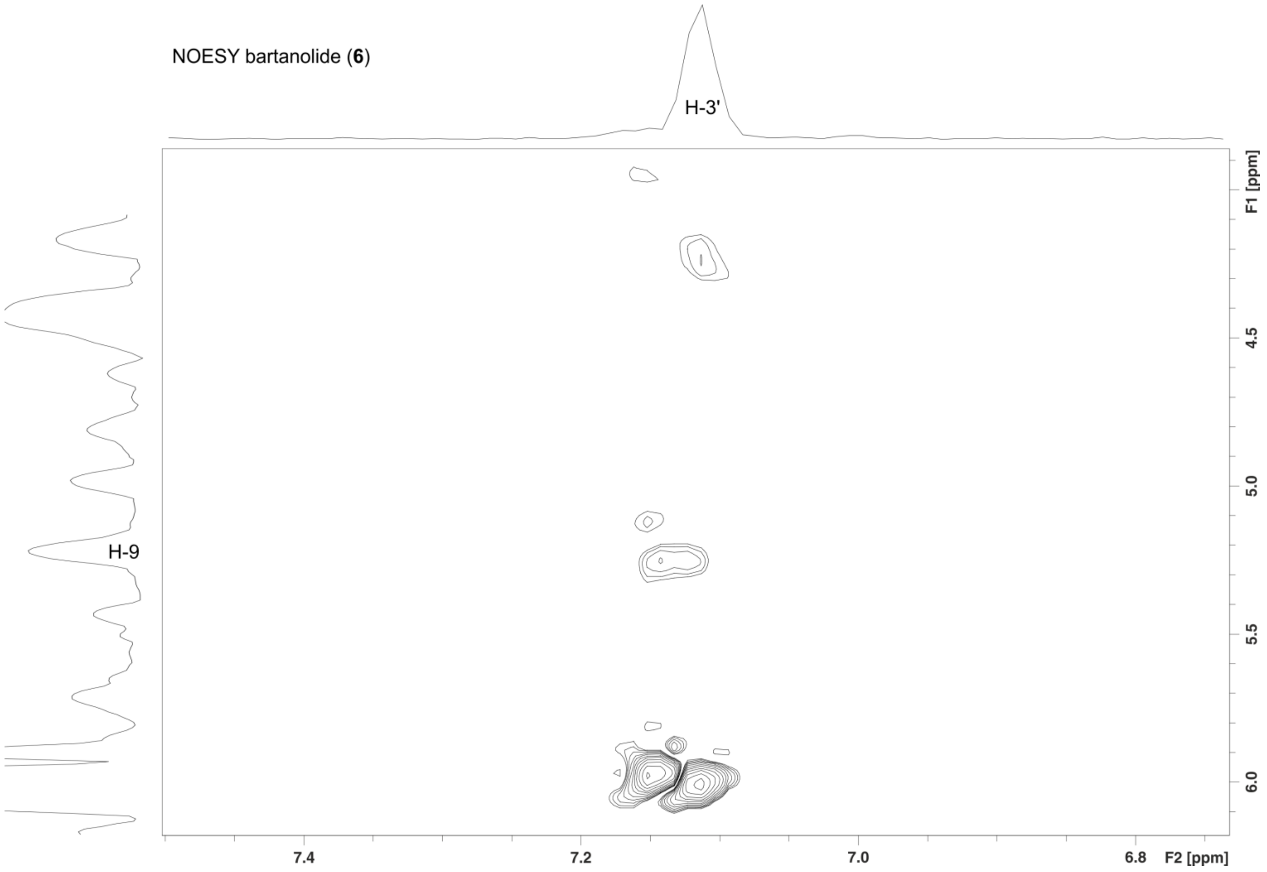

Supplement: Supplementary File 1 — Supplementary Information (PDF, 2042 KB) [file marinedrugs-11-00800-s001.pdf]
